# A Mouse Model Uncovers LKB1 as an UVB-Induced DNA Damage Sensor Mediating CDKN1A (p21^WAF1/CIP1^) Degradation

**DOI:** 10.1371/journal.pgen.1004721

**Published:** 2014-10-16

**Authors:** Rosaura Esteve-Puig, Rosa Gil, Elena González-Sánchez, Joan Josep Bech-Serra, Judit Grueso, Javier Hernández-Losa, Teresa Moliné, Francesc Canals, Berta Ferrer, Javier Cortés, Boris Bastian, Santiago Ramón y Cajal, Juan Martín-Caballero, Juana Maria Flores, Ana Vivancos, Vicenç García-Patos, Juan Ángel Recio

**Affiliations:** 1 Animal Models and Cancer Laboratory, Vall d'Hebron Research Institute (VHIR), Hospital Universitari Vall d'Hebron, Barcelona, Spain; 2 Proteomic Laboratory Medical Oncology Research Program, Vall d'Hebron Institute of Oncology - VHIO, Hospital Universitari Vall d'Hebron, Barcelona, Spain; 3 Pathology Department, Hospital Universitari Vall d'Hebron, Barcelona, Spain; 4 Clinical Oncology Program, Vall d'Hebron Institute of Oncology - VHIO, Barcelona, Spain; 5 Department of Dermatology, University of California San Francisco, San Francisco, California, United States of America; 6 Animal Laboratory Unit, Barcelona Biomedical Research Park (PRBB), Barcelona, Spain; 7 Surgery and Medicine Department, Veterinary School, Universidad Complutense de Madrid, Madrid, Spain; 8 Cancer Genomics Group Translational Research Program, Vall d'Hebron Institute of Oncology - VHIO, Hospital Universitari Vall d'Hebron, Barcelona, Spain; 9 Dermatology Department, Hospital Universitari Vall d'Hebron, Barcelona, Spain; Baylor College of Medicine, United States of America

## Abstract

Exposure to ultraviolet (UV) radiation from sunlight accounts for 90% of the symptoms of premature skin aging and skin cancer. The tumor suppressor serine-threonine kinase *LKB1* is mutated in Peutz-Jeghers syndrome and in a spectrum of epithelial cancers whose etiology suggests a cooperation with environmental insults. Here we analyzed the role of LKB1 in a UV-dependent mouse skin cancer model and show that *LKB1* haploinsufficiency is enough to impede UVB-induced DNA damage repair, contributing to tumor development driven by aberrant growth factor signaling. We demonstrate that LKB1 and its downstream kinase NUAK1 bind to CDKN1A. In response to UVB irradiation, LKB1 together with NUAK1 phosphorylates CDKN1A regulating the DNA damage response. Upon UVB treatment, *LKB1* or *NUAK1* deficiency results in CDKN1A accumulation, impaired DNA repair and resistance to apoptosis. Importantly, analysis of human tumor samples suggests that *LKB1* mutational status could be a prognostic risk factor for UV-induced skin cancer. Altogether, our results identify LKB1 as a DNA damage sensor protein regulating skin UV-induced DNA damage response.

## Author Summary

Environmental insults are directly involved in cancer development. In particular, Ultraviolet (UV) radiation has been associated to the acquisition of different types skin cancer and premature skin aging. UV radiation causes modifications in the genetic material of cells (DNA) that if not repaired properly will lead to a mutated DNA (mutated genes) which might trigger the development of cancer. Understanding the molecular basis of the UV-induced DNA damage response is important to elucidate the mechanisms of skin homeostasis and tumorigenesis. Here we provide a UVB-induced skin cancer animal model showing that LKB1 tumor suppressor is also a DNA damage sensor. Importantly, the data suggest that reduced amounts of LKB1 protein in skin could be a risk factor for UV-induced skin carcinogenesis in humans.

## Introduction

Ultraviolet (UV) radiation represents the number one leading cause for skin cancer. UV radiation can cause genetic mutations to DNA that if not repaired can lead to skin cancer. Elucidation of the mechanisms involved in UV-induced DNA damage response is important to understand the human disease, its treatment and prevention.

LKB1/STK11 is a ubiquitously expressed and evolutionary conserved serine-threonine kinase. *LKB1* was first identified as a tumor suppressor gene through its association with the Peutz-Jeghers syndrome [1] and is involved in a number of biological processes such as cell cycle control [2], [3], cellular energy metabolism [4], [5] and cell polarity [6]. The sub-cellular localization and activity of LKB1 is controlled through its interaction with the STE20-related adaptor (STRAD) and the armadillo repeat-containing mouse protein 25 (Mo25) [7], [8], regulating the activity of at least 14 downstream kinases-related to the AMPK family [9] and also, phosphorylating other substrates including STRAD and PTEN [10], [11]. LKB1 is phosphorylated on at least 8 residues, and evidence suggests that LKB1 auto-phosphorylates itself on at least four of these, whereas the other four are phosphorylated by upstream kinases [10], [12]. Among these residues Thr-366 is conserved in mammalian, *Xenopus* and *Drosophila* LKB1, and is located on a C-terminal non-catalytic moiety of the enzyme [13]. ATR and ATM phosphorylate LKB1^Thr366^ in response to ultraviolet irradiation (UV) and γ-radiation respectively, suggesting a role for LKB1 in response to DNA damage [14]. Although its function in DNA damage response has not been elucidated, mutation of Thr-366 to Ala or Asp partially inhibits the ability of LKB1 to suppress cell proliferation and it does not affect the nuclear cellular localization of LKB1. Moreover, phosphorylation of LKB1 at Thr-366 does not directly regulate LKB1 kinase activity [13], [14]. In addition to this, it has been suggested that LKB1-AMPK signaling controls non-homologous end joining (NHEJ) contributing to genome stability [15].

*LKB1* appears to be mutated or inactivated in sporadic cancers whose spectrum of tumor types, suggest cooperation with exposure to environmental carcinogens. Thus, *LKB1* has been found mutated in non-small cell lung carcinomas [16], [17], head and neck squamous cell carcinoma (SCC), pancreatic cancer [18] and melanomas [19]. It should be noted that hemizygous loss of chromosome 19p, spanning the *LKB1* locus, is observed in many cancer types. This observation together with the data generated from mouse models suggests that LKB1 can behave as a haploinsufficient tumor suppressor [17], [20]. Indeed, *Lkb1* deficiency sensitizes mice to DMBA-induced skin and lung SCC [21], and its inactivation in the context of RAS pathway activation facilitates the expansion of melanoma prometastatic tumor cell subpopulations [22] and progression of lung adenomas into carcinomas [23].

Cyclin-dependent kinase inhibitor 1A (CDKN1A) has an important role modulating DNA repair processes, inhibiting cell cycle progression and apoptosis. It competes for PCNA binding with several PCNA-reliant proteins that are directly involved in DNA repair processes including mismatch repair (MMR), base excision repair (BER) and translesion DNA synthesis (TLS) [24]–[29]. Evidence also suggest that CDKN1A may regulate nucleotide excision repair (NER), although its exact role has been controversial [30]. It has been showed that CDKN1A is proteolytically degraded in response to low-dose UV radiation by a mechanism that requires the physical interaction of CDKN1A with PCNA [31], [32]. Furthermore, the ability to degrade CDKN1A under this condition is critical for optimal DNA repair and to preserve genomic stability [24], [33]–[35].

The *Hgf* (hepatocyte growth factor) transgenic mouse (*Hgf* ^Tg^) is a useful experimental model for determining the consequences and elucidating the mechanisms of exposure to UV radiation [36]–[38]. Here, we show that *LKB1* haploinsufficiency sensitizes *Hgf* ^Tg^ mouse to UVB-induced skin cancer through a mechanism that involves CDKN1A protein accumulation. Interestingly, LKB1 and its downstream kinase NUAK1 bind and phosphorylate CDKN1A contributing to its physiological regulation. LKB1 deficiency leads to CDKN1A accumulation in response to UVB radiation, promoting both defects in DNA repair and protection from apoptosis. Our findings suggest that the mutational status of LKB1 can serve as a novel risk factor for UV-induced skin tumors.

## Results

### *Hgf* ^Tg^; *Lkb1* ^+/−^ mice are highly prone to neonatal UVB-induced SCCs

**Figure 1:**
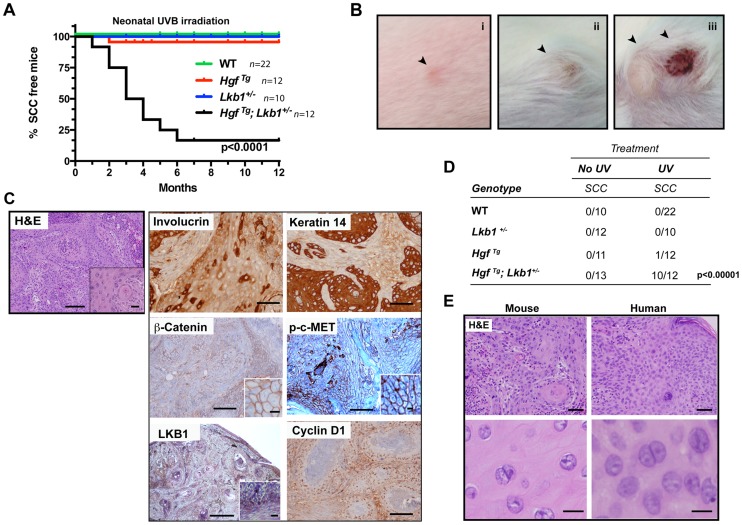
*Hgf^Tg^; Lkb1^+/−^* mice are highly prone to neonatal UVB-induced SCCs. (**A**) Kaplan–Meier analysis of neonatal UVB irradiated wild type (WT), *Hgf^Tg^, Lkb1^+/−^* and *Hgf* ^Tg^; *Lkb1* ^+/−^ mice documenting the development of SCC. *Hgf^Tg^, Lkb1^+/−^* mice showed significant differences in UVB-induced tumor development, P<0.0001). (**B**) (i to iii), gross image and progression of SCC in an *Hgf* ^Tg^; *Lkb1* ^+/−^ mouse after UVB irradiation. (**C**) Histology of cutaneous SCC. Hematoxilin-Eosin staining of mouse tumor samples and immunostaining of SCC for involucrin keratin-14, β-catenin, p-C-MET, LKB1 and cyclin D1. Bars 200 µm, Inset bar 50 µm. (**D**) Penetrance of skin-SCC in neonatal UVB-irradiated vs. non-irradiated mice. *P*-value was calculated using a fisher's exact test between UVB-irradiated vs. non-irradiated mice. (**E**) Hematoxilin-Eosin staining of mouse and human samples showing histological similarities. Bars upper panels 150 µm, bars lower panels 50 µm.

We previously demonstrated that LKB1 is involved in HGF signaling [4]. However, its *in vivo* role in response to UVB radiation has not been assessed. We examined the role of LKB1 in suppression of UVB-induced skin cancer using the HGF transgenic mouse model [38] by generating the *Hgf* ^Tg^; *Lkb1* ^+/−^ mouse. Exposure of neonatal mice (3.5 days old) to a single suberythemal dose of UVB radiation was sufficient to induce robust development of skin tumors only in *Hgf* ^Tg^; *Lkb1* ^+/−^ mouse (Figure 1A). Early lesions appeared on the albino FVB background as persistent, discolored spots between 3 and 4 weeks of age, giving rise to frank and ulcerated tumors with a median onset age of 45 days (Figure 1A and B (i, ii, iii)). Histologic analysis and staining for Involucrin, Cytokeratin-14 and β-Catenin revealed that these skin tumors were all malignant SCC (Figure 1C). Tumors also showed high amounts of p-c-MET as an indication of HGF activity, were positive for Cyclin D1 and showed a heterogeneous staining of LKB1 (Figure 1C). *Hgf* ^Tg^; *Lkb1* ^+/−^ mice also showed an altered tumor spectrum relative to either *Lkb1* ^+/−^ or *Hgf* ^Tg^ mice (Figure S1A). Ten out of twelve UVB-irradiated *Hgf* ^Tg^; *Lkb1* ^+/−^ mice developed SCCs. Tumors did not appear in non-irradiated animals or irradiated wild type or *Lkb1* ^+/−^ animals, and just one irradiated *Hgf* ^Tg^ mouse out of twelve developed an SCC (Figure 1D). As expected four *Hgf* ^Tg^ irradiated mice developed three nevi and one melanoma, albeit these mice were over 12 months old (Figure S1A). The tumor incidence in the UVB-irradiated *Hgf* ^Tg^; *Lkb1* ^+/−^ mice was 83% showing variable multiplicity between animals (Figure S1B and C). Hence, *Lkb1* heterozygosity in an *Hgf* ^Tg^ background sensitizes mice to single-dose UVB-induced skin SCC.

### UVB-induced tumors are undifferentiated and resemble human SCCs, but do not progress from papillomas

Histopathological examination of mouse tumors revealed a remarkable similarity to lesions found in SCC patients. Human and mouse tumor SCC samples showed atypical proliferative keratinocytes forming irregular nests invading the stroma. These anastomosing growths of cords and nests were composed of cells that have nuclear atypia with irregular, large nuclei with one or more nucleoli and abundant eosinophilic cytoplasm. Mitotic figures are noted occasionally.(Figure 1E). Papillomas were rarely observed prior to SCC development in serially monitored UVB-induced *Hgf* ^Tg^;*Lkb1* ^+/−^ mice, and we did not detect papillomatous changes adjacent to carcinoma in our histologic analyses. Finally, the incidence of papillomas (1 of 25 mice) was comparable in the wild type and single mutant cohorts (2 of 23 *Hgf* ^Tg^ mice and 1 of 22 *Lkb1* ^+/−^ mice developed papillomas) (Figure S1B). Consistent with this and the lack of papilloma-SCC progression, no *H-Ras* mutations were detected in the UVB-induced SCC arising in the *Hgf* ^Tg^; *Lkb1* ^+/−^ mice. However, these tumors showed high levels of p-c-Met that activates RAS and PI3K pathways. Tumors also exhibited undifferentiated and malignant regions characterized by a decrease in the expression levels of LKB1, β-Catenin, E-Cadherin and α6-Integrin (Figure S1D). In agreement with the high tumor growth rate, the proliferation markers cyclin D1 and Ki67 (Figure 1C and S1E) indicated that these tumors were highly proliferative. They also showed low levels of apoptosis measured by counting cleaved caspase-3 positive cells (Figure S1E). In agreement with previous studies [20] and the heterogeneous LKB1 tumor staining, LKB1 was not expressed in SCC primary tumor-derived cell lines (Figure S1F), suggesting that the *Lkb1* wild-type allele (Figure S1G) could be inactivated by multiple mechanisms in SCC, including deletion and possibly point mutation or promoter hypermethylation.

### *Lkb1* deficiency leads to the accumulation of CDKN1A in response to UVB-induced DNA damage

We next investigated mice skin integrity. Immunohistochemical analysis of Cytokeratin-14, E-Cadherin and β-Catenin revealed comparable staining in the epidermis of wild type, *Hgf* ^Tg^, *Lkb1* ^+/−^, and *Hgf* ^Tg^; *Lkb1* ^+/−^ mice, indicating that keratinocyte differentiation is not compromised neither with the half genetic dose of LKB1 nor overexpression of HGF (Figure S2A). As expected, skin of *Hgf* ^Tg^ and *Hgf* ^Tg^;*Lkb1* ^+/−^ mice showed high levels of p-c-Met and based on p-Erk1/2 staining, an increased activation of the RAS pathway (Figure S2A). Ki67 staining indicated that in response to UVB irradiation (2 h and 48 h post irradiation) a large number of keratinocytes in the epidermal basal layer of *Lkb1* ^+/−^ and *Hgf* ^Tg^; *Lkb1* ^+/−^ mice were recruited into cell cycle (Figure S2B). *Hgf* ^Tg^; *Lkb1* ^+/−^ mice also demonstrated aberrantly dividing cells in the epidermal suprabasal layers and evidence for the lose of cell division polarity (Figure S2B).

Since UVB-irradiation triggered skin tumorigenesis, we quantified the number of basal keratinocytes showing elevated levels of p-CHK2 after UVB irradiation, as an indicator of DNA damage. Two hours post-irradiation *Hgf* ^Tg^ and *Lkb1* ^+/−^ mice did not show significant differences in the number of p-CHK2 positive cells (Figure S3A).

**Figure 2:**
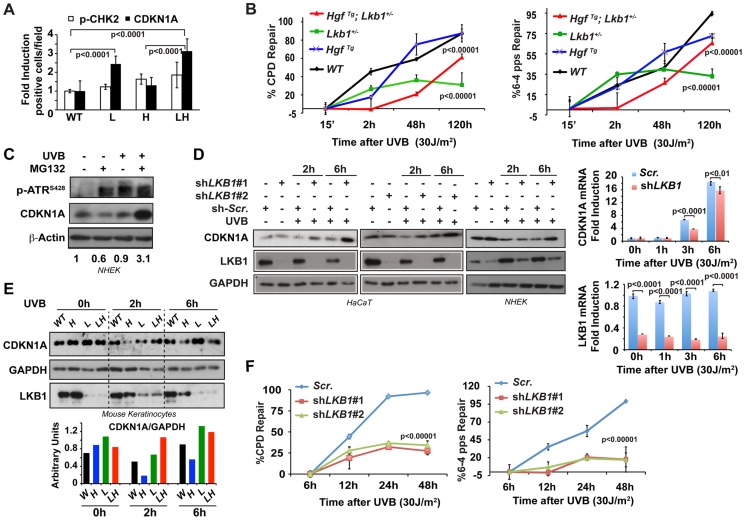
*Lkb1* haploinsufficiency induces CDKN1A accumulation after UVB-mediated DNA damage. (**A**) Representation of the average amounts of p-CHK2 and CDKN1A in the skin of mice from different genotypes at 48 h post irradiation. (WT, *Lkb1* ^+/−^ (L), *Hgf* ^Tg^ (H) and *Hgf* ^Tg^; *Lkb1* ^+/−^ (HL)). *P*-values were calculated performing a student's *t*-test. (**B**) Global genomic UVB-induced DNA repair analysis performed in skin DNA from WT *Lkb1^+/−^* and *Lkb1^+/−^; Hgf^Tg^* mice. Graphs show the average repair at different time point. At least five mice per genotype and time point were analyzed. Error bars represent mean ± SD. *P*-values were calculated performing a student's *t*-test. (**C**) CDKN1A degradation is induced after UVB DNA damage in Normal Human Epidermal Keratinocytes (NHEK). NHEK were pretreated for 2 h with MG132 (200 nM) and treated with UVB (30 J/m^2^). Western-blot shows the level of p-ATR, CDKN1A, LKB1. β-Actin is shown as a loading control. One representative experiment of three is shown. (**D**) Depletion of LKB1 in normal human epidermal keratinocytes (NHEK) and immortalized normal keratinocytes (HaCat cells) induced the accumulation of CDKN1A after UVB irradiation. Cells stably infected with lentiviral constructs expressing either scramble shRNA (*Scr*.) or two different shRNAs sequences against human *LKB1* (*shLKB1#1, shLKB1#2*). Western blot show the amount of LKB1, CDKN1A and GAPDH after UVB treatment (30 J/m^2^). (*n* = 3 experiments). On the right panel, LKB1 depletion does not induce the transcriptional up regulation of *CDKN1A*. *CDKN1A* and *LKB1* mRNA abundance were determined after UVB irradiation (30 J/m^2^) by qRT-PCR. Error bars represent mean ± SD. Measurements were normalized against *18S* mRNA and *GAPDH* (*n* = 3 experiments). (**E**) UVB irradiation induces CDKN1A accumulation in *Lkb1^+/−^* and *Hgf* ^Tg^; *Lkb1* ^+/−^ mice. Isolated keratinocytes from different mouse skin genotypes were UVB irradiated (30 J/m^2^) (WT, *Lkb1* ^+/−^ (L), *Hgf* ^Tg^ (H) and *Hgf* ^Tg^; *Lkb1* ^+/−^ (HL)). Western blot shows the amount of LKB1, CDKN1A and GAPDH at the indicated time points post-irradiation. Graph show the normalized quantification of bands. (**F**) Global genomic UVB-induced DNA repair analysis. HaCat cells infected with scrambled shRNA (Scr.), *shLKB1#1* or *shLKB1#2* were irradiated with (30 J/m^2^). Graphs show the quantification of the modification's repair normalized by the amount of DNA from at least three independent experiments. Error bars represent mean ± SD. *P*-value was calculated doing a student's *t*-test.

It is known that CDKN1A plays an important role in DNA repair [24], [30], [31]. In response to low doses of UV-irradiation CDKN1A is proteolytically degraded by a mechanism that requires the physical interaction of CDKN1A with PCNA [39], [40] allowing the recruitment of PCNA to the damaged DNA regions and optimal DNA repair [35]. Interestingly, *Lkb1* ^+/−^ and *Hgf* ^Tg^; *Lkb1* ^+/−^ mice, showed an atypical response to UVB irradiation, presenting a significant accumulation of CDKN1A in basal keratinocytes in response to UVB-induced DNA damage (Figure S3B). Thus, although there were small differences in the total number of cells damaged among the different genotypes, there was a significant accumulation of CDKN1A in *Lkb1* ^+/−^ and *Hgf* ^Tg^; *Lkb1* ^+/−^ mice (P<0,0001 WT *vs. Lkb1* ^+/−^; or WT *vs. Hgf* ^Tg^; *Lkb1* ^+/−^) (Figure 2A), suggesting a DNA damage repair deficiency upon *Lkb1* haploinsufficiency. In fact, a global genomic DNA repair analysis [41] of mouse skin confirmed that *Lkb1* ^+/−^ and *Hgf* ^Tg^; *Lkb1* ^+/−^ mice had significant UVB-induced DNA damage repair deficiencies (*Lkb1* ^+/−^ mice repair 30% of cyclobutane pyrimidine dimers (CPD) and 31.25% of 6-4 photoproducts (6-4pps) relative to WT mice; *Hgf* ^Tg^; *Lkb1* ^+/−^ mice repair 65% of CPD and 68% of 6-4pps relative to WT mice) (Figure 2B and S3C). Hence, UVB irradiation in the context of *Lkb1* haploinsufficiency leads to the accumulation of CDKN1A and impaired DNA repair.

### LKB1 mediates CDKN1A degradation in response to UVB damage

Next we sought to determine the molecular mechanism(s) that underlie the response to UVB-induced DNA damage. CDKN1A proteolytic degradation after low doses of UV is known to be critical for PCNA release and optimal DNA repair [24], [31], [32]. Indeed, pretreatment of normal human keratinocytes with the proteasome inhibitor MG132 induced the accumulation of CDKN1A in response to UVB irradiation (Figure 2C) evidencing the fine-tune regulation of CDKN1A amounts upon low doses of UVB irradiation. To investigate the role of LKB1 in response to UVB irradiation regulating CDKN1A protein levels, we knocked down (mRNA) *LKB1* in wild type immortalized keratinocytes and in normal human epidermal keratinocytes (NHEK). In the absence of LKB1, UVB irradiation induced the accumulation of CDKN1A (Figure 2D and S3D) together with PCNA (Figure S3E). qRT-PCR analysis demonstrated that UVB-induced CDKN1A accumulation in the absence of LKB1 was not due to its transcriptional up-regulation. In agreement with the previously described role of LKB1 regulating CDKN1A expression [3], [42], [43], *LKB1* knockdown cells showed a significant decrease in the UVB-induced transcriptional regulation of CDKN1A (Figure 2D). Moreover, the total amounts of LKB1 decreased overtime in response to UV irradiation (Figure 2D). Accumulation of CDKN1A in response to UVB was also observed in mouse keratinocytes generated from *Lkb1* ^+/−^ and *Hgf* ^Tg^; *Lkb1* ^+/−^ animals compared to cells isolated from wild type and *Hgf* ^Tg^ mice (Figure 2E). We next investigated whether CDKN1A accumulation in *LKB1* knockdown cells interfered with the repair of the UVB-damaged DNA. A global genomic DNA repair assay [41] showed that parental cells fully repair specific UVB-induced DNA damage 48 h after irradiation. However, two different clones of *LKB1* knockdown cells repaired 35% and 20% of CPDs and 6-4pps, respectively, at the same time point (Figure 2F and S3F). Thus, these new evidence support the role of LKB1 in UVB-induced DNA damage repair, regulating the amount of CDKN1A protein. Altogether, these data suggested that the UVB-induced DNA damage response mediated by CDKN1A stability and/or transcriptional regulation was compromised in the context of *Lkb1* haploinsufficiency.

### LKB1 binds to CDKN1A

**Figure 3:**
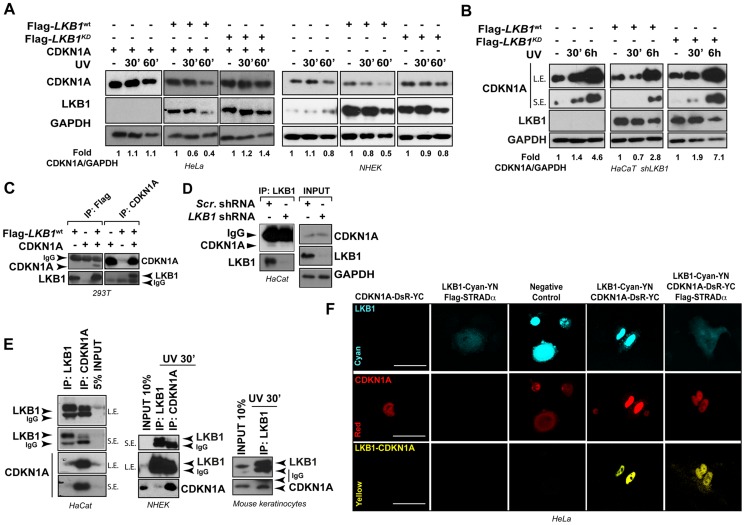
LKB1 binds CDKN1A. (**A**) LKB1 kinase activity is necessary to induce UVB-mediated CDKN1A degradation. HeLa cells and Normal Human Epidermal Keratinocytes (NHEK) were transfected with either Flag-*Lkb1* ^WT^ or Flag-*Lkb1* ^KD^ together with Flag-*STRADα*, *Mo25α* and *CDKN1A* where indicated. Western-blot shows the abundance of CDKN1A and LKB1, at different time points in response to UVB irradiation (30 J/m^2^). GAPDH is shown as a loading control. One representative experiment out of three is shown. (**B**) *LKB1* Knockdown HaCaT cells were transfected and treated as in (**A**). Western blots show amount of CDKN1A, LKB1 and GAPDH at the indicated time points in response to UVB irradiation (30 J/m^2^). GAPDH is shown as a loading control. (*n* = 3 experiments) (**C**) 293T cells were transfected with equimolar amounts of Flag-*Lkb1* ^WT^ Flag-*STRADα* and *Mo25α* and/or *CDKN1A*. Western-blots show the amount of LKB1 or CDKN1A after immunoprecipitation with anti-Flag or anti-CDKN1A antibodies (*n* = 3 experiments). (**D**) Western-blot shows the amounts of endogenous CDKN1A bound to LKB1 in HaCat cells infected with scrambled shRNA or *shLKB1#1*. (**E**) Western-blot showing the amounts of CDKN1A bound to LKB1 in HaCat cells, NHEK and Normal Mouse Keratinocytes. Either 5% or 10% of the total lysate is shown as a control. A short (S.E.) and a long exposure (L.E.) are shown. (*n* = 3 experiments). (**F**) Bimolecular Fluorescence Complementation assay (BiFC), *Lkb1*-*CFP*-*YFPN* and *CDKN1A-mRFP1-YFPC* constructs were transfected in HeLa cells, in the absence or presence of Flag-*STRADα* and *MO25α*. Two non-interacting nuclear proteins A-C-YN and B-R-YC are shown as negative control. One representative experiment out of three is shown.

We next investigated whether LKB1 kinase activity was necessary for UVB-induced CDKN1A degradation. We reconstituted the system in HeLa cells (deficient for LKB1) and expressed the different LKB1 isoforms (wild type LKB1 or LKB1^KD^ (kinase dead)) in normal human epidermal keratinocytes (NHEK). Expression of CDKN1A together with either wild type LKB1 or LKB1^KD^ (kinase dead) in HeLa cells showed that in response to UVB radiation there was an accumulation of CDKN1A in LKB1^KD^ transfected cells, suggesting that LKB1 kinase activity was involved in the regulation of CDKN1A protein amounts in response to UVB irradiation. Similar response was observed in NHEK transfected cells (Figure 3A). Additionally, expression of mouse wild type *Lkb1* but not the kinase dead mutant *Lkb1* ^KD^ in *LKB1* knocked down HaCaT cells (HaCaT *shLKB1*), promoted the UVB-induced degradation of CDKN1A (Figure 3B).

Interestingly, LKB1 and CDKN1A form part of the same immunocomplexes (Figure 3C). This association was also observed with the endogenous proteins at basal levels and in response to UVB radiation (Figure 3D and E) and it appeared to be specific to CDKN1A since CDKN1B (p27), a related CDK inhibitor family member, did not bind to LKB1 (Figure S4A). LKB1 and CDKN1A protein-protein interaction was confirmed by Bimolecular Fluorescence Complementation (BiFC) [44] (Figure 3F and Figure S4B). Construction of two different *LKB1* mutants lacking C-terminal 20 (*Flag-LBK1^Δ416^*) and 113 (*Flag-LBK1^Δ323^*) amino acids, showed that carboxy-terminal region of LKB1 (Figure S4C) seemed to be involved in the binding to CDKN1A and, in a lesser extent to HSP90, a known LKB1 binding protein (Figure S4C). Altogether, these results suggest that LKB1 physically interacts with CDKN1A immunocomplexes and its kinase activity is involved in the CDKN1A UVB-induced degradation.

### LKB1 and its downstream kinase NUAK1 phosphorylate CDKN1A

**Figure 4:**
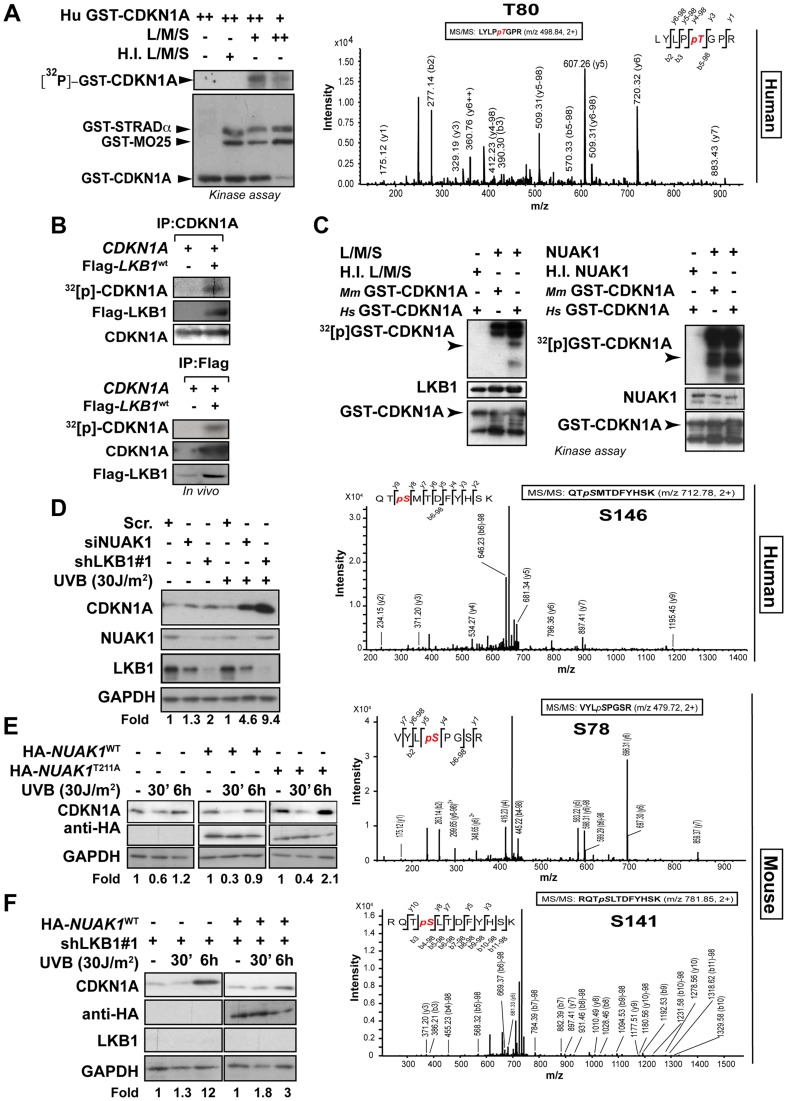
LKB1 and NUAK1 phosphorylate CDKN1A. (**A**) In vitro kinase assay using recombinant heterotrimer His-LKB1/GST-STRADα/GST-Mo25α and recombinant human *Hs*-GST-CDKN1A. Autoradiography shows in vitro phosphorylation of CDKN1A by *LKB1*. Western-blot shows the loading for GST-tagged proteins. Assays were performed in triplicates. Mass spectrometry analysis of *in vitro* phosphorylated CDKN1A by LKB1. (**B**) LKB1 phosphorylates CDKN1A in vivo. 293T cells were transfected with CDKN1A and/or equimolar amounts of Flag-*Lkb1* ^WT^, Flag-*STRADα* and *MO25α*. After in vivo labeling with [^32^P], CDKN1A and Flag-LKB1 were immunoprecipitated and the amount of CDKN1A phosphorylated determined by autoradiography. Western blots show the immunoprecipitated CDKN1A and LKB1 in one representative experiment out of three. (**C**) In vitro kinase assay using recombinant heterotrimer His-LKB1/GST-STRADα/GST-Mo25α, NUAK1 and recombinant human (*Hs*) and mouse (*Mm*) GST-CDKN1A. Below, mass spectrometry analysis of *in vitro* phosphorylated CDKN1A by LKB1 or NUAK1. One out of four experiments is shown. (**D**) HaCat cells were transfected with scrambled (Scr. shRNA), *LKB1* (shRNA) or *NUAK1* (siRNA). Western-blot shows the amounts of CDKN1A, LKB1 and NUAK1. GAPDH is used as loading control. (**E**) HaCat cells were transiently transfected with either HA-*NUAK1* ^WT^, HA-*NUAK1* ^T211A^ and treated with UVB for the indicated time points. Amounts of CDKN1A and NUAK1 proteins are showed. GAPDH is the loading control. (**F**) HaCat cells stably infected with *shLKB1* were transfected with HA-*NUAK1* and treated with UVB for the indicated times. Variation of the amount of CDKN1A was assessed by western-blot. GAPDH is shown as loading control.

Next, we investigated the functional consequences of this interaction and examined whether LKB1 was able to phosphorylate CDKN1A. *In vitro* kinase assays using recombinant His-LKB1/GST-STRADα/GST-Mo25 heterotrimeric complex and recombinant human GST-CDKN1A demonstrated that LKB1 phosphorylates CDKN1A (Figure 4A). Mass spectrometry analysis of the phosphorylated CDKN1A identified Thr80 as the residue phosphorylated by LKB1 *in vitro* (Figure 4A and S5A). *In vivo* labeling of cells with [^32^P]-orthophosphate followed by the immunoprecipitation of CDKN1A revealed that CDKN1A becomes phosphorylated in the presence of LKB1, STRADα and Mo25α Furthermore under these conditions CDKN1A bound to LKB1 immunocomplexes was also phosphorylated (Figure 4B). However, sequence alignment analysis of mouse, rat and human CDKN1A revealed that Thr80 is not conserved in mouse and rat proteins. Instead, mouse and rat proteins exhibit a Serine at position 78 not existing in the human orthologue (Figure S5B). Thus, we investigated whether LKB1 or any of its downstream AMPK family kinases were involved in the regulation of mouse CDKN1A. Results showed that LKB1 only phosphorylates human CDKN1A at Thr80 and not mouse CDKN1A, however, NUAK1, a downstream kinase of LKB1, phosphorylated human CDKN1A at Thr146 and mouse CDKN1A at Ser78 and Thr141, the equivalent residues in human CDKN1A (Thr80 and Thr146, respectively) (Figure 4C). Although, LKB1 *in vitro* phosphorylation of human CDKN1A (0.32±0.08 pmol [^32^P]/pmol protein) was less efficient than phosphorylation of a known substrate such as AMPKα (1,2±0.11 pmol [^32^P]/pmol protein), both, mouse and human CDKN1A were efficiently phosphorylated *in vitro* by NUAK1 (0.8±0.18 pmol [^32^P]/pmol protein and 0.9±1.2 pmol [^32^P]/pmol protein, respectively) (Figure S5C). We identified by mass spectrometry phosphorylation of Ser78 in endogenous CDKN1A upon UVB irradiation in mouse melanoma cells (Figure S5D), Phosphorylation on Ser78 was significantly decreased in LKB1 depleted cells (30% *vs.* 1% of peptide phosphorylated respectively; p<0.0001) (Figure S5E). In agreement with the role of LKB1 and NUAK1 regulating CDKN1A degradation upon UVB irradiation, non-phosphorylable human CDKN1A mutants T80A, S146A and double mutant T80A;S146A were accumulated after UVB treatment as compared to the wild type protein. Interestingly, mutation of both residues (T80A;S146A) caused a synergistic accumulation compared to the single mutations (Figure S6A).

Besides the low amounts of NUAK1 within HaCat cells, we found NUAK1 and CDKN1A form part of the same immunocomplexes (Fig. S6B). Depletion of NUAK1 partially reproduced the accumulation of CDKN1A in response to UVB observed in the absence of LKB1 (Figure 4D and Figure S6C), and induced phosphorylation of CDKN1A Ser146 upon UVB radiation was absent in NUAK1 knockdown cells (Figure S6C). Moreover, expression of mutant HA-NUAK1^T211A^ that cannot be activated by LKB1, led to the accumulation of CDKN1A, upon UVB treatment (Figure 4E) and expression of NUAK1 in LKB1 depleted cells almost totally reconstituted the normal response to UVB (Figure 4F). Altogether these results show evidence indicating that LKB1 and its downstream kinase NUAK1 phosphorylate CDKN1A and are involved in its regulation in response to UVB radiation.

### UVB-induced phosphorylation of LKB1^T366^ mediates CDKN1A degradation

**Figure 5:**
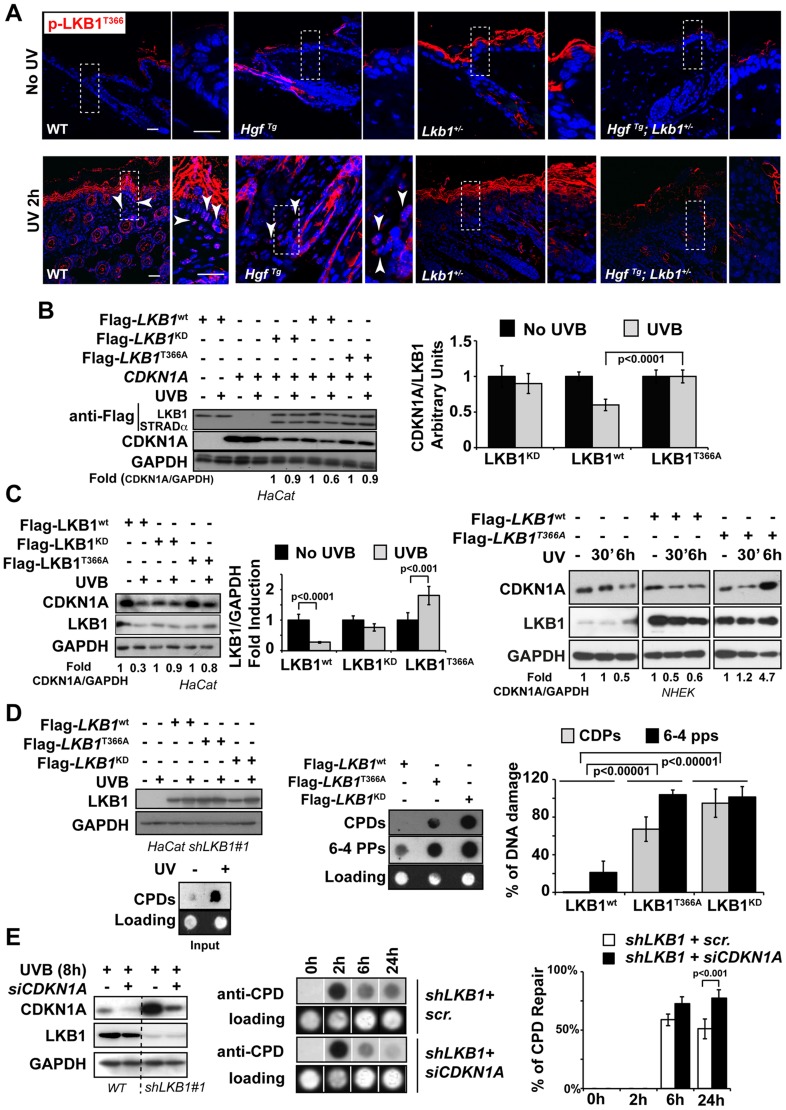
UVB-induced phosphorylation of LKB1^T366^ mediates CDKN1A degradation and DNA repair. (**A**) Mouse skin from non irradiated and UVB irradiated mouse were stained with anti p-LKB1^T366^ antibody. Dashed squares indicate amplified areas. Bar represent 100 µm. (**B**) HeLa cells were transfected with *CDKN1A*, *MO25α*, Flag-*STRADα* and either Flag-*Lkb1* ^WT^, Flag-*Lkb1* ^KD^ or Flag-*Lkb1* ^T366A^ mutant and treated with UVB (30 J/m^2^) and lysed 30 min after UVB irradiation. Western-blot shows the expression of LKB1, STRADα and CDKN1A. Graph shows the quantifications of the bands normalized against GAPDH. One representative experiment out of three is shown. (**C**) On the left HaCat cells were transfected either with Flag-*Lkb1* ^WT^, Flag-*Lkb1* ^KD^ or Flag-*Lkb1* ^T366A^ together with Flag-*STRADα* and *Mo25α*. Western-blot shows the amount of LKB1 and endogenous CDKN1A 30 min after UVB irradiation (30 J/m^2^). Graph show quantifications under the different conditions. (n = 3 experiments). Error bars represent mean ± SD. *P*-value was calculated performing a student's *t*-test. On the right NHEK were transfected with either Flag-*Lkb1* ^WT^ or Flag-*Lkb1* ^T366A^ mutant and treated with UVB (30 J/m^2^) and lysed at the indicated time points. Western-blot shows the amount of LKB1 and endogenous CDKN1A. Fold induction of CDKN1A expression normalized against GAPDH is showed. One representative experiment out of three is shown. (**D**) Global UVB-induced DNA damage repair assay. LKB1 knockdown HaCat cells were transfected and treated as in (**C**). Graphs show normalized quantification (n = 3 experiments) of DNA damage repair 48 h after UVB irradiation. *P*-value was calculated performing a student's *t*-test. (**E**) HaCat cells and *LKB1* HaCat knockdown cells were transiently transfected with *CDKN1A* siRNA and treated with UVB (30 J/m^2^). Western-blot shows the amounts of CDKN1A. Graph shows percentage of UVB-induced DNA damage repair.

It has been suggested that LKB1 plays a role in genotoxic stress [45], [46]. However the molecular mechanism(s) involved are not fully understood. LKB1^T366^ becomes phosphorylated in response to UV irradiation [14] (Figure S7A and B). We observed that LKB1^T366^ was phosphorylated more efficiently in the skin of WT and *Hgf* ^Tg^ mouse than in the skin of *Lkb1* ^+/−^ and *Hgf* ^Tg^; *Lkb1* ^+/−^ animals (Figure 5A). In a reconstituted system LKB1 wild type promoted the degradation of CDKN1A in response to UVB, however, LKB1^T366A^ and LKB1^KD^ mutants did not promote this effect (Figure 5B). Analysis of CDKN1A immunocomplexes from the same samples showed that LKB1^T366A^ mutant has a diminished affinity for CDKN1A (Figure S7C). We identified PCNA as part of the immunocomplex, supporting the role of LKB1 in DNA damage response. In response to UVB the number of PCNA molecules bound to CDKN1A decreased in Flag-*Lkb1^WT^* transfected cells, while in Flag-*Lkb1^T366A^* transfected cells were unmodified (Figure S7C). The effect of Flag-*Lkb1* ^T366A^ mutant on CDKN1A stability in response to UVB, was also partially observed with endogenous protein (Figure 5C), and the number of phospho-LKB1^T366^ molecules recruited to the CDKN1A immunocomplexes increased in response to UVB (Figure S7D). Moreover, expression of mutant Flag-*Lkb1* ^T366A^ also impaired the cells ability to repair UVB-induced DNA damage supporting the role of LKB1 and CDKN1A degradation in DNA repair (Figure 5D). In agreement to this, depletion of CDKN1A in UVB-irradiated LKB1 knockdown cells allows them to repair DNA more efficiently (Figure 5E) Thus, these results suggest that UVB-induced phosphorylation of LKB1^T366^ regulates CDKN1A stability, which is linked to the response to UVB-induced DNA damage repair.

### Lack of LKB1 protects from UVB-induced apoptosis

**Figure 6:**
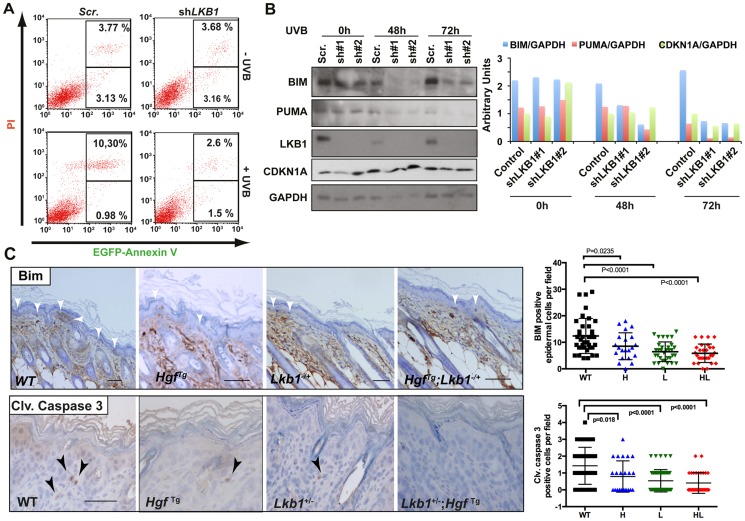
Loss of LKB1 and accumulation of CDKN1A in response to UVB contributes to keratinocyte transformation and resistance to UVB-induced apoptosis. (**A**) HaCat cells infected either with scrambled or *shLKB1* were irradiated with UVB (30 J/m^2^). Then, at 48 h, EGFP-Annexin V and PI (propidium iodide) positive cells were analyzed by flow cytometry. Histograms show the result from FACS analysis. (**B**) Time course of UVB irradiated (30 J/m^2^) HaCat cells infected either with scrambled or two different *shLKB1* (#1 and #2). Western-blot shows the amounts of BIM, PUMA, CDKN1A and LKB1. GAPDH is shown as loading control. Graph shows quantification of bands normalized by GAPDH. (**C**) Immunohistochemistry showing Bim and cleaved caspase-3 staining in wild type (WT), *Lkb1* ^+/−^ (L), *Hgf* ^Tg^ (H) and *Hgf* ^Tg^; *Lkb1* ^+/−^ (HL) mice. Bar represent 100 µm. Graphs show quantification of positive cells in mouse skin (at least 25 fields (20×)/genotype). Bars represent mean values. *P*-values were calculated using a student's t-test.

Treatment of *Hgf* ^Tg^; *Lkb1* ^+/−^ mice with a single neonatal dose of UVB radiation led to the development of SCC. Under LKB1 haploinsufficiency, UVB treatment promoted an accumulation of CDKN1A followed by a deficiency in DNA damage repair. In addition to the suggested role of CDKN1A in response to low doses of UV-induced DNA damage, accumulation of CDKN1A after different genotoxic insults protect cells from apoptosis [47]–[49]. Thus, we investigated the consequences of *LKB1* loss in UVB-induced apoptosis. As expected LKB1 behaved as a tumor suppressor [3]. Knockdown of *LKB1* in HaCat cells increased proliferation and cells (cell-cell) contact inhibition (Figure S7E). UVB irradiation induced the accumulation of CDKN1A in the absence of LKB1. We noticed that there was a significant (P<0.001) higher number of viable cells after UVB irradiation in *LKB1* depleted cells than in parental cells (Figure S7F). In agreement to this *LKB1* knockdown cells were significantly (P<0.001) more resistant to UVB-induced apoptosis than parental cells (2.8% *LKB1* knockdown cells *vs.* 10.3% parental cells at 48 hours post-irradiation (Figure 6A). Resistance to apoptosis correlated with lower amounts of pro-apoptotic proteins BIM and PUMA (Figure 6B). Knockdown cells also showed lower amounts of CDKN1A at 72 h than parental cells (Figure 6B). This result was also observed in skin keratinocytes of *Lkb1* ^+/−^ and *Hgf* ^Tg^; *Lkb1* ^+/−^ mice compared to wild type animals at 72 h–80 h post-irradiation.(P = 0.004 and P<0.0001 respectively) (Figure S8A) Moreover, *Lkb1* ^+/−^ and *Hgf* ^Tg^; *Lkb1* ^+/−^ mice showed significant lower amounts of keratynocytes staining positive for Bim and cleaved-Caspase 3 at 48 hours post-irradiation, than wild type mice (P<0.0001, Figure 6C). Thus, the data show evidence supporting the contribution of the loss of LKB1 and accumulation of CDKN1A to UVB-induced apoptosis resistance, leading to malignancy.

### Loss of LKB1 expression is an early event in human SCC

**Figure 7:**
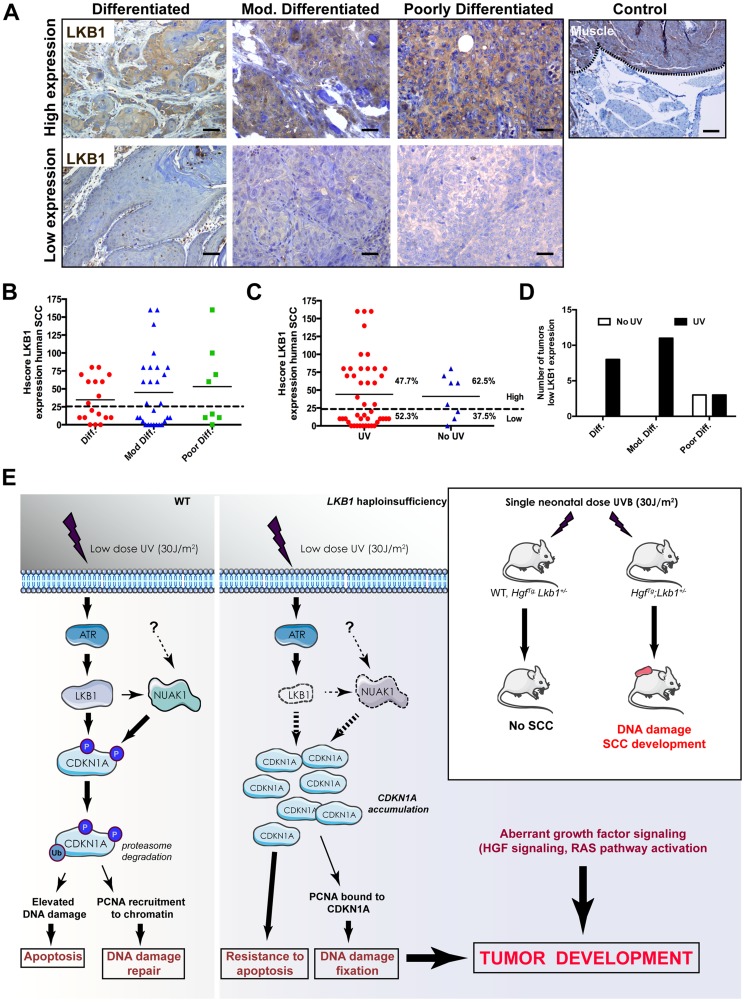
LKB1 expression in human skin-SCC. (**A**) Representative images of differentiated, moderately differentiated and poorly differentiated human skin SCC, showing high expression and low expression amounts of LKB1. A positive control of LKB1 specific staining (Muscle) is shown. (**B**) Distribution of human tumor samples (n = 51) according to their stage of differentiation and the Hscore for LKB1. (**C**) Distribution of the same samples in respect to the exposure of the samples to UV according to their anatomical distribution. (**D**) Distribution of low LKB1 expression samples within the different tumor stages and according to their UV status. (**E**) Model for the role of LKB1 in UVB-induced DNA damage response. In response to low doses of UV radiation, LKB1 becomes phosphorylated by ATR and induces CDKN1A degradation through its phosphorylation liberating PCNA and its recruitment to chromatin for DNA repair. In the absence of LKB1, CDKN1A is not phosphorylated and accumulates, contributing to UV-induced mutagenesis and resistance to apoptosis. According to the animal model this UVB-induced DNA damage cooperates with aberrant growth factor signaling for tumor development.

To evaluate the relevance of LKB1 in human skin SCC we examined the expression of LKB1 by immunohistochemistry in 54 human skin SCC samples (Table S1). Samples were comprised of anatomical localizations compatible with UV-exposed and non-UV-exposed regions. Roughly 50% of the samples showed either very low or no staining for LKB1 (Figure 7A and B). We found that the lack of expression of LKB1 was independent of the differentiation stage of the tumor samples (*n* = 18 differentiated, *n* = 30 moderately differentiated and *n* = 8 poorly differentiated) (Figure 7B). Interestingly, there was a tendency where samples showing low or no staining for LKB1 localized preferentially in UV-exposed areas (52,3% of (n = 45) vs. 33,3% in non-UV-exposed areas (n = 9)) (Figure 7C). Moreover, samples from UV exposed areas and low LKB1 expression amounts felt into any tumor stage category, while all samples from non-UV exposed areas and low expression of LKB1 were poorly differentiated (Figure 7D). Interestingly, analysis of a curated data set of 225 tumors from another relevant UV-induced skin tumor such as cutaneous melanoma (c-Bioportal, MSKCC) [50] showed alterations in LKB1 or NUAK1 in 22.2% of cases that were mutually exclusive (odds ratio 0.625 (no association); 95% Confidence Interval: 0.138438–2.821652; *P*-*value*: 0.412752 (Fisher's Exact Test)). In fact staining of human skin tumor SCC samples with LKB1 and NUAK1 showed an inverse Hscore correlation (95% confidence interval, *P* = 0.0033) between LKB1 and NUAK1 expression (Figure S8B). This mutual exclusivity of LKB1 or NUAK1 alterations is observed other tumor types including head and neck squamous cells carcinomas (19.7% of data set from 295 tumors), (95% Confidence Interval: 0.552751–5.723118 *P*-value: 0.250381 (Fisher's Exact Test), cervical squamous cell carcinoma (30.6% of data set from 36 tumors), (95% Confidence Interval: 0.095179–10.506562 *P*-value: 0.695155 (Fisher's Exact Test) and lung squamous cell carcinoma (15.3% of data set from 177 tumors), (95% Confidence Interval: 0.109684–7.534791 *P*-value: 0.703561 (Fisher's Exact Test).

Hence, this additional data suggest that the loss of LKB1 expression at early stages could contribute to UV-induced skin cancer development (Figure 7E).

## Discussion

Genotoxic environmental insults such as UV are associated with the development of skin cancer. DNA damage repair has been proven to be crucial in fending off detrimental effects such as mutagenesis and cell death. Here, we show that LKB1 tumor suppressor is a DNA damage sensor, and together with its downstream kinase NUAK1, contributes to UVB-induced CDKN1A degradation, allowing DNA repair and genomic integrity.

*LKB1*/STK11 is mutated in sporadic human cancers whose spectrum of tumor types suggests cooperation with exposure to environmental carcinogens [17]–[19]. In humans, skin-SCC is associated with chronic rather than intermittent intense exposure to UV radiation [51]. However, under conditions of *LKB1* haploinsufficiency in an *Hgf* ^Tg^ background, a single neonatal suberythemal dose of UVB was sufficient to induce skin-SCC bypassing the papilloma-SCC sequence. This result highlights the *in vivo* role of LKB1 in response to genotoxic insults, in particular to UVB irradiation. In contrast to human SCC samples, in mice we did not detect any mutations in *HRAS*. This could be the reason why our mice do not develop papillomas. However, it is likely that the requirement for RAS pathway activation for tumor development and progression in humans is achieved in the mouse through the activation of c-MET by HGF over-expression. Most of the SCC tumors showed a heterogeneous expression of LKB1. In this matter, lack of expression of LKB1 was observed often associated to undifferentiated tumor regions and in mouse tumor-derived cell lines. Hence, contrary to the previously published DMBA-induced SCC mouse model [21] and in agreement with the benign gastrointestinal polyposis associated with *Lkb1* deficiency; in our model malignant SCC pathogenesis seems not to require biallelic inactivation of *Lkb1*.

It is known that ATM/ATR are important kinases involved in DNA damage response [52]. Previous work suggested that a yet unidentified kinase would be likely to be involved acting between ATR and CDKN1A in response to low doses of UV irradiation [24]. CDKN1A is induced after ionizing radiation, but degraded after UV exposure [24]. Thus, UV-induced degradation of CDKN1A is necessary for optimal DNA repair and to preserve genomic stability. This is accomplished by CDKN1A ubiquitylation and degradation via the CRL4(Cdt2) ubiquitin ligase complex, setting free proliferating cell nuclear antigen (PCNA) from the CDKN1A-PCNA complexes and controlling translesion DNA synthesis [24], [35], [39], [53]. In agreement with previous publications our results show that lack of LKB1 compromised the transcriptional regulation of CDKN1A (Figure 2D) [43], however, it also promoted accumulation of CDKN1A protein in response to UVB irradiation. We show that LKB1 deficiency impedes physiological UVB-induced CDKN1A degradation, impairing DNA damage repair and consequently contributes to mutagenesis and tumor development. It is known that, multitask Ser/Thr kinase LKB1 becomes phosphorylated by ATR at Thr366 in response UV [14]. However, the physiological role for this modification is unknown. We show that mutation of LKB1 Thr-366 to Ala impaired the cells ability to repair UVB-induced DNA damage by affecting CDKN1A UVB–induced degradation. Furthermore, in humans, LKB1 and its downstream kinase NUAK1 bind and phosphorylate CDKN1A (at Thr80 and Ser146, respectively) contributing to its degradation in response to UVB and DNA repair. Although LKB1 it is known to phosphorylate AMPK family members, the amount of pmol of phosphate incorporated per pmol of CDKN1A compared head to head to the *in vitro* efficiency toward AMPK, suggested its capability to phosphorylate other substrates different to the AMPK family members. In this matter, NUAK1 was very effective phosphorylating both human and mouse CDKN1A. Of note is that, Thr80 is not conserved in mouse CDKN1A sequence, instead, there is a Ser at position 78. Interestingly, NUAK1 phosphorylates mouse CDKN1A at Ser78 and Ser141, the homologous residues in the human orthologue, and also conserved in the rat protein. Although, the data suggest that NUAK1 contribution is mediated by LKB1, these results do not exclude its LKB1-independent effect. In fact NUAK1 has been previously involved in DNA damage response phosphorylating p53 and participating in the transcriptional regulation of *CDKN1A* promoter [45]. We hypothesize that this redundancy in humans (LKB1 and NUAK1) compared to mouse (NUAK1) provides biological robustness to a mechanism involved in a UV genotoxic response. This could be particularly relevant to humans which skin is clearly more exposed to environmental insults such as UV radiation.

Several lines of evidence support the biological role of LKB1 in DNA damage response. First, LKB1 becomes phosphorylated at Thr366 in response to UVB. Second, LKB1 kinase activity seems to be necessary for CDKN1A degradation in response to UVB radiation. Third, LKB1 binds and phosphorylates CDKN1A. Fourth, NUAK1, the LKB1 downstream kinase, rescues the LKB1 knockdown phenotype in response to UVB. Fifth, LKB1 binds to CDKN1A and upon UVB treatment there is an increased association of CDKN1A molecules to phopho-LKB1^T366^. Furthermore, LKB1^T366A^ mutant has a diminished binding to CDKN1A compared with LKB1^WT^ and LKB1^KD^ mutant, it does not promote CDKN1A degradation in response to UVB radiation, and impairs DNA damage repair. In addition to all these, there is concomitant degradation of LKB1 and CDKN1A in response to UV. Although, we cannot fully explain this observation, it is tempting to speculate that these two molecules are simultaneously proteasome-degraded, permitting the liberation of PCNA and DNA repair. The later, is also supported by the increment in UVB-induced DNA damage repair in LKB1 depleted cells when CDKN1A is knocked down. Although the link between CDKN1A degradation and DNA repair has been extensively demonstrated and our data, and other recent work [35] confirm this connection, how UVB-induced CDKN1A phosphorylation leads to its degradation and whether the concomitant LKB1 degradation is connected needs to be further investigated.

From the pathogenic point of view in addition to the UVB induced mutations, the loss of LKB1 tumor suppressor would also contribute to deregulate cell proliferation and cell-to-cell contact inhibition. Furthermore, LKB1 deficient cells were resistant to UVB-induced apoptosis, probably through the accumulation of CDKN1A [47]–[49]. Altogether this would ultimately favor the fixation of UVB-induced mutations and tumor development. All these data suggest that in humans silencing a single copy of *LKB1* would be sufficient to increase the risk of the acquisition and accumulation of UV-induced mutations, placing LKB1 as an important player in response to environmental insults associated to the acquisition of skin cancer. Indeed, analysis of human samples showed that 50% of skin-SCC lack or showed very low amounts of LKB1 expression. The absence of expression of LKB1 was independent of the differentiation stage of the tumor and had a tendency to be more frequent in SCC from UV–exposed areas. This suggests that the loss of LKB1 expression is an early event in tumor development and/or progression. Since our animal model demonstrates that *LKB1* haploinsufficiency is sufficient to cause the accumulation of UVB-induced DNA damage, we posit that the mutational status of *LKB1* is a prognostic risk factor for UV-induced skin cancers. In agreement to this, in melanoma and squamous cell carcinomas, *LKB1* is mutated in 2% and 11% of tumor samples, respectively (COSMIC-Wellcome Trust Sanger Institute). Furthermore, our data and results from other studies (c-Bioportal, MSKCC) show that tumors with a clear environmental component including, melanoma, head an neck squamous cell carcinoma, lung squamous cell carcinoma and endometrial squamous cell carcinoma, alterations in LKB1 or NUAK1 are mutually exclusive, reinforcing the role of this molecular axis in DNA damage and genomic instability.

In summary, here we unveil a novel role for LKB1 as a UV-induced DNA damage sensor protein. Reduced amounts of LKB1 are enough to impair UVB-induced DNA repair and cooperate with HGF signaling to promote skin cancer. At the molecular level the results indicate that we have identified the missing link between ATR and the physiological regulation of CDKN1A in response to UVB. In this matter, following UVB irradiation LKB1 becomes phosphorylated by ATM/ATR and then, LKB1 and its downstream kinase NUAK1 phosphorylate CDKN1A contributing to its physiological regulation. Thus, deficiencies in LKB1 promotes fixation of UVB–induced mutations, resistance to UVB-induced apoptosis contributing to tumor development.

## Materials and Methods

### Mouse strains and UV treatment

*Hgf* ^Tg^ and *Lkb1* ^+/−^ strains and UV treatment have been previously described [20], [38]. Data from our survival analysis was performed using Prism 6 (GraphPad Software Inc.). All animal work have been conducted according to relevant national and international guidelines and approved by the Animal Ethics Committee from the Institution (Institut de Recerca Vall d'Hebron (Barcelona, Spain).

### Reagents, cell culture, expression vectors, antibodies, lentiviral infection and transfections

293T, HeLa and HaCat cells were obtained from ATCC. NHEK (Normal juvenil Human Epidermal Keratinocytes) were obtained from Promo-Cell (Heilderberg, Germany) and cultured in Keratinocyte growth medium 2 (Promo-Cell). Mouse keratinocytes were isolated as described in [54] MG132 was from Sigma-Aldrich (Saint Louis, MO, USA) C_f_ = 200 nM. γ–^32^P-P-ATP and γ–^32^P-Orthophosphate were purchased from PerkinElmer (Waltham, Massachusetts, USA). Plasmids pCMV5-human *CDKN1A*, pCMV5-human *CDKN1A* T80A and pCMV5-human *CDKN1A* T80D were generated using QuickChange Site-Directed Mutagenesis (Stratagene, Cedar Creek, TX, USA). pCMV5-Flag-mouse-*Lkb1^WT^* and pCMV5-Flag-mouse-*Lkb1^KD^* (kinase dead) were a generous gift from D. Alessi, Univ. Dundee, UK; pCMV5-Flag-mouse-*Lkb1^T366A^* was generated using Quick-Change Site-Directed Mutagenesis (Stratagene, Cedar Creek, TX, USA). pcDNA4-Flag-*STRADα* and pKCFP-*MO25α* were a gift from M. Sanchez-Céspedes (PEBC-IDIBELL, Barcelona, Spain). p*EYFP*-*p27^wt^* was a gift from G. Mills (MD Anderson Cancer Center, Houston, USA). For LKB1 silencing five different lentiviral pLKO.1-shLKB1 constructs were obtained from Sigma-Aldrich (Saint Louis, MO, USA). For *NUAK1* and *CDKN1A* siRNA were purchased from Invitrogen. All transfections and lentiviral infections were performed as described [4]. All pCMV5-Flag-mouse-*Lkb1* isoforms were co-transfected with equimolar amounts of pcDNA4-Flag-*STRADα* and pKCFP-*MO25α*. Total amount of transfected DNA was compensated using an empty vector (E.V.). Constructs were transfected into cells with Lipofectamine 2000 Transfection Reagent (Invitrogen), following the manufacturer's recommended protocol. Immunoprecipitation was performed in RIPA buffer using M2-agarose (Sigma-Aldrich) 24 h post-transfection and after UVB treatment.

### Antibodies

Keratin-14, Involucrin, E-cadherin, β-catenin and α6-Integrin were obtained from HG. Palmer, VHIO, Spain, while LKB1 (D60C5), phosphor-ERK1/2 and total ERK1/2, phospho-Met (Tyr1234/1235), phospho-ATR (Ser428), cleaved caspase-3, PUMA, Bim and phospho-CHK2 antibodies were from Cell Signaling (Danvers, MA USA). Ki67 was from Master Diagnostica (Granada, Spain). G3PDH (GAPDH) was from Trevigen Inc. (Gaithersburg, MD USA). Cyclin D1, p27 (C-19), anti-HA (Y-11), and LKB1 were from Santa Cruz (Santa Cruz, CA, USA). Anti –NUAK1 was from Proteintech (Proteintech Group, Inc. Chicago, IL, USA). Anti–BIM for IHC-P was from Thermo scientific (Thermo Fisher Scientific Inc., Waltham, MA USA). Anti-CDKN1A Ab-11 (Clone CP74) was from Thermo Fisher Scientific (Runcorn, Cheshire, UK). Anti–PCNA was from Abcam, (Cambridge, UK). Phospho-LKB1 (T366) was purchased from MRC, University of Dundee, Glasglow, UK. β-Actin was from Millipore, Madrid, Spain. Secondary antibodies included Alexa Fluor 488, Alexa Fluor 563 (Invitrogen, Carlsbad, CA USA), anti-rabbit and anti-mouse linked to horseradish peroxidase (GE Healthcare, Barcelona, Spain), MOM kit, and ABC Vector kit (Vector-Labs, Burlingame, CA, USA).

### Irradiation and cell extracts and immunoblots

Cells were irradiated with UVB (30 J/m^2^) at 50%–70% confluency without medium nor the lid. After treatments, cells were lysed in RIPA buffer and immuno-blots were performed as previously described [4].

### Cell cycle analysis, cell viability and apoptosis assays

Has been performed as previously described in [4], [55].

### Immunohistochemistry and immunofluorescence

Paraffin-embedded tumor samples were subjected to immunocytochemistry according to the manufacturer's antibody protocol. The samples used in this Project were provided by the Tumor Bank of the Vall d'Hebron University Hospital Biobank with appropriate ethical approval (supported by the Xarxa de Bancs de Tumors de Catalunya sponsored by Pla Director d'Oncología de Catalunya (XBTC); supported by the RETICS de Biobancos (ISCIII). All cases were evaluated independently by an expert dermatopathologist (BF) and one trained Molecular Biologist (JHL) blinded for patient groups, taking into account the percentage of positive cells and intensity of the staining, which was assessed semiquantitatively. Final results were obtained utilizing the average of the two values. Whenever a major discrepancy was observed between both observers, the cases were discussed using a multi-headed microscope. LKB1 was evaluated using Histoscore (Hscore) there was calculated: Hscore = (1× % weak staining cells)+(2× % moderate-strong staining cells) with results ranging from 0 to 200. Samples with an Hscore<25 were classified as low expression samples.

### Bimolecular Fluorescence Complementation (BiFC) assay

pCMV-*CDKN1A*-mRFP1-YFP-C and pCMV-*LKB1*-CFP-YFP-N constructs were generated, introducing wild type human *LKB1* (EMBL-EBI: AF035625) and human *CDKN1A* (EMBL-EBI: L25610) sequences into pCMV-R-YC and pCMV-C-YN vectors (obtained from Brack-Werner, Institute of Molecular Virology, GSF-National Research Center for Environment and Health, Neuherberg, Germany) [44]. HeLa cells were transiently transfected with these constructs for 24 h and YFP, CFP and mRFP1 fluorescence was analyzed by confocal microscopy (Espectral FV1000 Olympus).

### Global genomic DNA repair assay

Cells were harvested 0, 24, 48 or 72 h after UV irradiation. Unirradiated control cells were also harvested. Genomic DNA was isolated using the DNeasy kit (Qiagen Mississauga, Ontario) according to the manufacturer's protocol. DNA (100 ng in 0.5 M NaOH and 10 mM EDTA) was denatured by boiling for 10 min. Ice-cold ammonium acetate (2 M) was added to a final concentration of 1 M. Denatured DNA was spotted onto a nitrocellulose membrane pre-wetted with 6× SSC using a slot-blot apparatus (Bio-Dot SF, Bio-Rad, Mississauga, Ontario). The filter was baked at 80°C for 2 h. Thymine dimers were quantified using the monoclonal antibody MC-062 (clone KTM53, Kamiya Biomedical, Seattle, WA). Bound antibody was detected by ECL plus (Amersham, Baie d'Urfè, Quèbec), and quantified by autoradiography. The membrane was re-probed with radiolabeled mouse genomic DNA to quantify the amount of the sample DNA per slot. The antibody signal was normalized to the amount of DNA per lane, and the rate of lesion removal was calculated [41] and graphed.

### Kinase assays, *in vivo* labeling and measurement of p21 phosphorylation compared to AMPK kinase

*In vitro* LKB1 (Millipore) kinase protein assay were performed as described in [8] using recombinant human *Hs*-GST-CDKN1A (Abcam) or mouse *Mm*-GST-CDKN1A. *In vivo* γ–^32^P metabolic labeling was described in [56]. Quantification of kinase activities was done as in [10].

### Liquid chromatography-mass spectrometry analysis

Samples were separated on a 10% SDS-PAGE gel, and the gel stained with colloidal Coomassie blue. Protein bands of interest were processed as described in [57].

## Supporting Information

Figure S1 Related to Figure 1. *Hgf* ^Tg^; *Lkb1* ^+/−^ mice are highly prone to neonatal UVB-induced SCCs. (**A**) Table showing tumor spectrum described in UVB -irradiated and Non UVB-irradiated mice. (**B**) Graph showing the number UVB-irradiated mice developing skin tumors. *Hgf* ^Tg^ (H), *Lkb1* ^+/−^ (L) and *Hgf* ^Tg^; *Lkb1* ^+/−^ (HL). (**C**) Multiplicity of skin-SCC in neonatal UVB-irradiated mice, *Hgf* ^Tg^ (H), *Lkb1* ^+/−^ (L) and *Hgf* ^Tg^; *Lkb1* ^+/−^ (HL). UVB-Induced SCCs are highly proliferative with low apoptosis rates and present undifferentiated regions. (**D**) Immunohistochemistry of UVB-induced SCCs showing representative staining of Cyclin D1, Involucrin, Keratin-14, p-c-MET, β-Catenin (Bars 200 µm), and LKB1 (Bar 500 µm). Insets show a detail of the staining (Bars 50 µm). A panel of mouse SCCs showing differentiated (Diff.) and undifferentiated (Undiff.) regions. Immunohistochemistry shows staining for LKB1, β-Catenin (Bars 500 µm),inset (Bars 100 µm), E-Cadherin (Bars 100 µm) and α6-Integrin (Bars 600 µm). Inset at show the loss of α6-Integrin expression in one of the keratinocyte nests (Bar 200 µm) (**E**) Ki67 and cleaved caspase-3 staining of three different tumors (Bars 200 µm). (**F**) Western-Blot showing the amount of LKB1 and β-Actin in primary mouse SSC cell lines derived from tumors raised on *Hgf* ^Tg^; *Lkb1* ^+/−^ mice. HaCat cells total lysates are used as a control. (**G**) Western-Blot showing the amount of LKB1and CDKN1A in skin extracts from indicated mice. GAPDH is showed as loading control.
(TIF)

Figure S2 Relate to Figure 1. (**A**) Keratynocytes differentiation is not compromised neither in the absence of LKB1 or overexpression of HGF. Mouse skin form different genotypes were stained for K14 (a–d),E-Cadherin (e–h), β-Catenin (i–l), p-Erk1/2 (n–p) and p-c-Met (q–t). Representative images are shown. Bars are 400 µm from a–d and j–m; 200 µm e–h, n–q and r–u. (**B**) *Lkb1* ^+/−^ and *Hgf* ^Tg^; *Lkb1* ^+/−^ mice showed an increased number of keratynocytes recruited into the cell cycle upon UVB irradiation. Bars are 400 µm and 100 µm for magnifications Graphs show quantification of Ki67 positive cells per field 2 hours and 48 hours after UVB irradiation (30 J/m2). At least 30 field/point were evaluated. Error bars represent mean ± SD. P-values were calculated doing a student's t-test.
(TIF)

Figure S3 Related Figure 2. Lkb1 haploinsufficiency induces CDKN1A accumulation after UVB-mediated DNA damage. (**A**) Representative images of mouse skin stained with anti p-Chk2 antibody 48 h after UVB irradiation. Bars 100 µm. Graph shows quantification of p-CHK2 positive basal keratinocytes 48 h post-irradiation. At least fifty fields (20×) from each different mouse genotype (n = 10) were quantified (WT, *Lkb1* ^+/−^ (L), *Hgf* ^Tg^ (H) and *Hgf* ^Tg^; *Lkb1* ^+/−^ (HL)). P-values were calculated using a student's t-test. Error bars represent mean ± SD. (**B**) Immunohistochemistry of CDKN1A staining showing representative images of mouse skin non-irradiated and 48 h after UVB irradiation. Bars 100 µm. Quantification of CDKN1A positive cells in mouse skin 48 h post-irradiation. At least two hundred and fifty fields (20×) per mouse genotype (n = 10) were quantified. Bars represent mean values. P-values were calculated using a student's t-test. (**C**) Representative dot blot showing a Global genomic UVB-induced DNA repair analysis performed in skin DNA from WT, *Lkb1* ^+/−^ and *Hgf* ^Tg^; *Lkb1* ^+/−^ mice. Briefly, spotted DNA on the membrane was probed against anti CPD and anti 6-4pps antibodies, as described in experimental procedures. Quantifications of the analysis of at least five mice per genotype and time point are shown in Figure 2 B. (**D**) Immunofluorescence of CDKN1A in parental infected with Scr. scramble shRNA and LKB1 knocked down HaCat cells (shLKB1#1). One representative experiment out of three is shown. Cells were treated with 30 J/m2 of UVB radiation. Dapi shows nuclear staining. Bars 50 µm. (**E**) PCNA co-localizes with CDKN1A in the nucleus in response to UVB radiation in LKB1 knockdown cells. HaCat cells and stable LKB1 knockdown cells were treated with UVB (30 J/m2). Then, 6 hours after treatment cells were stained for PCNA and CDKN1A. Dapi was used for nuclear staining. Representative images are shown. Bars = 50 µm. (**F**) Representative dot blot showing a global genomic UVB-induced DNA repair analysis performed in HaCat cells infected either with scrambled shRNA (Scr.) or two different shRNA against LKB1 (shLKB1#1 and shLKB1#2) and UVB irradiated (30 J/m2). Briefly, spotted DNA on the membrane was probed against anti CPD and anti 6-4pps antibodies, as described in experimental procedures. Quantifications of three independent experiments at different time points are shown in Figure 2E.
(TIF)

Figure S4 Related to Figure 3. (**A**) LKB1 binds to CDKN1A but not to CDKN1B (p27). HeLa cells were transfected with equimolar amounts of Flag-Lkb1WT, Flag STRADα and Mo25 together with either CDKN1A or CDKN1B-EYFP (p27). Western-blot shows the presence of CDKN1A and CDKN1B proteins bound to LKB1. Total lysates are shown for transfection control. (**B**) Constructs for BiFC assay, Lkb1-CFP-YFPN and CDKN1A-mRFP1-YFPC constructs were transfected in HeLa cells, in the absence or presence of Flag-STRADα and MO25α (figure 3D). (**C**) Schematic representation of LKB1 deletion mutants. HeLa cells were transfected with either the different LKB1 constructs together with CDKN1A. LKB1 was immunoprecipitated using anti Flag antibody. After SDS-PAGE the amount of CDKN1A bound to LKB1 was assessed by western-blot. HSP90 is showed as a positive control binding protein for LKB1.
(TIF)

Figure S5 Related to figure 4. LKB1 mediates mouse CDKN1A phosphorylation in response to UVB. (**A**) In vitro kinase radioactive assay of LKB1:STRAD:MO25 (250 ng) using human recombinant GST-CDKN1A and GST-CDKN1AT80A mutant as a substrate (300 ng). Number below shows differences in fold changes in GST-CDKN1A phosphorylation. (**B**) Mouse and Human sequence alignment. Human T80 and Mouse S78 appear highlighted. (**C**) In vitro kinase assay of LKB1:STRAD:MO25 (250 ng) using AMPKα/β/γ as a substrate (300 ng). Pmol of phosphate incorporated per pmol of substrate by either LKB1 or NUAK1 is shown in the graph. All the experiments were performed in triplicates. Error bars represent mean± SD. (**D**) 37-31T2 mouse melanoma cells were treated with MG132 (200 nM) for 2 h and then irradiated with UVB (30 J/m2). Cells were lysed six hours post irradiation. Western-blot showing the accumulation of CDKN1A. Then CDKN1A was immunoprecipitated. Gel shows CDKN1A amounts after coomassie blue staining. Bands corresponding to CDKN1A size were analyzed by mass spectrometry. Fragmentation spectrum of mouse protein peptide containing S78 is shown. (**E**) 37-31T2 cells infected with scrambled (Scr.) or shLkb1 were treated as in (**D**) followed by mass spectrometry of immunoprecipitated CDKN1A percentage of non-phosphorylated and phosphorylated peptides at residue S78 is shown in the graph. Error bars represent mean ± SD. P-value were calculated using a student's t-test.
(TIF)

Figure S6 Related to figure 4. CDKN1A phosphorylation site mutants T80A, S146A and T80A; S146A are accumulated in responses to UVB irradiation. (**A**) HaCat cells were transiently transfected with wild type and mutant isoforms of CDKN1A. Then cells were UVB irradiated (30 J/m2) and lysed after 30 minutes and 6 h. Western blot shows the amounts of CDKN1A, LKB1 and GAPDH. Graph shows normalized quantification against GAPDH. One representative experiment out of three is showed. NUAK1 and CDKN1A form part of the same immunocomplexes. 37-31T2 mouse melanoma cells were treated with MG132 (200 nM) for 2 h and then irradiated with UVB (30 J/m2). Cells were lysed six hours post irradiation. (**B**) Two different antibodies against p21WAF1/CIP1were used to immunoprecipitate CDKN1A at the indicated dilutions. Western-blots show the amount of CDKN1A immunoprecipitated and the amount of NUAK1 present in the immunocomplexes. (**C**) HaCat cells transiently transfected with either NUAK1 siRNA or scrambled siRNA and HaCat cells stable infected with shLKB1 were irradiated with 30 J/m2 of UVB. Total protein lysates were analyzed by SDS-PAGE 6 h post-irradiation. Amounts of p-CDKN1ASer146, p21WAF1/CIP, NUAK1, LKB1 and GAPDH are shown. Graphs show the amounts of p-CDKN1ASer146 relative o the amount of CDKN1A and the amounts of CDKN1A relative to the amount of GAPDH. P- values were calculated performing a student's t-test.
(TIF)

Figure S7 Related to figure 5. UVB-induced phosphorylation of LKB1T366 is involved in the binding to CDKN1A. (**A**) Representative pictures (n = 3 experiments) of immunofluorescence of p-LKB1T366 in HaCat cells 4 h after UVB irradiation. Dapi shows nuclear staining. (**B**) HaCat and 293T cells were irradiated with 30 J/m2 of UVB (n = 3 experiments). Samples were analyzed by western-blot at the times indicated. The amount of p-LKB1T366 relative to the amount of LKB1 is shown. Quantification of p-LKB1T366 relative to the amount of LKB1 in the time course is shown. (**C**) Total lysates from (Figure 5 A) were used to immunoprecipitate CDKN1A. Western-blot shows the amount of Lkb1 and PCNA in the immunocomplexes. Graphs on the right show the quantification of LKB1 and PCNA bound to CDKN1A (n = 3 experiments). (**D**) CDKN1A was immunoprecipitated from HaCat cells transfected either with scrambled shRNA or shLKB1#1 6 h after UVB irradiation (30 J/m2). Western-blot shows the abundance of p-LKB1 bound to CDKN1A. Graph shows the ratio of p-LKB1T366 bound to CDKN1A under the different conditions. One representative experiment out of three is shown. Error bars represent mean ± SD. P-value was calculated performing a student's t-test. Related to Figure 6. Depletion of LKB1 promotes pro-tumorigenic features and resistance to UVB radiation. (**E**) HaCat cells stably infected with shLKB1 showed an increased proliferation and lost cell-cell contact inhibition. Representative images using one of the three different shLKB1 are shown. Bars are 200 µm. (**F**) HaCat cells infected either with scrambled or shLKB1 were irradiated with UVB (30 J/m2). Representative images of cells stained against CDKN1A 10 h post-irradiation are shown. On the right number of viable and dead cells were quantified at different time points by nuclear staining exclusion (Guava-ViaCount).
(TIF)

Figure S8 (**A**) Expression of CDKN1A in mouse skin after 72–80 h after UVB irradiation. Quantification of positive cells of at least 20 fields (20×) per genotype (three different mice) is showed on the right. Bar represent 100 µm. (**B**) Staining of same tumor samples for LKB1 and NUAK1. Correlation of the LKB1 and NUAK1 HScore for each sample is plotted on the right. For this plot sample from patient #5 staining negative for both proteins was excluded. 95% confidence interval is showed in the graph. Bar represent 200 µm.
(TIF)

Table S1 Related to Figure 6. Human tumor samples used in the study. Diagnostic, grade of differentiation (staging), Hscore, anatomical localization and UV exposure component are shown.
(TIF)
