# Supplementary figures and images for: A Mouse Model Uncovers LKB1 as an UVB-Induced DNA Damage Sensor Mediating CDKN1A (p21WAF1/CIP1) Degradation
Source: PLoS Genet. 2014 Oct 16;10(10):e1004721. doi: 10.1371/journal.pgen.1004721 (PMC4199501; doi:10.1371/journal.pgen.1004721)

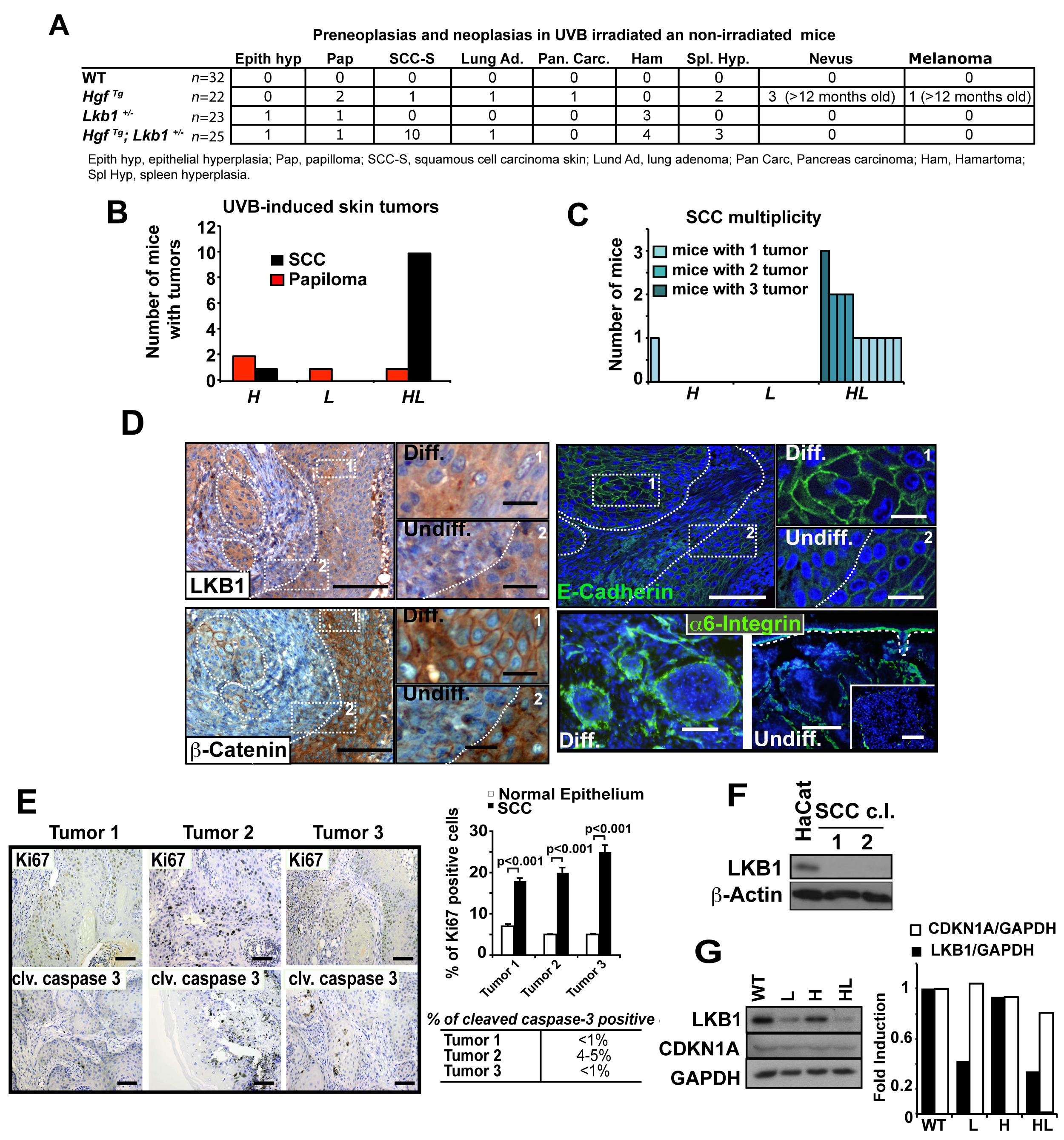

Supplement: Figure S1 — Related to Figure 1. Hgf Tg; Lkb1 +/− mice are highly prone to neonatal UVB-induced SCCs. (A) Table showing tumor spectrum described in UVB -irradiated and Non UVB-irradiated mice. (B) Graph showing the number UVB-irradiated mice developing skin tumors. Hgf Tg (H), Lkb1 +/− (L) and Hgf Tg; Lkb1 +/− (HL). (C) Multiplicity of skin-SCC in neonatal UVB-irradiated mice, Hgf Tg (H), Lkb1 +/− (L) and Hgf Tg; Lkb1 +/− (HL). UVB-Induced SCCs are highly proliferative with low apoptosis rates and present undifferentiated regions. (D) Immunohistochemistry of UVB-induced SCCs showing representative staining of Cyclin D1, Involucrin, Keratin-14, p-c-MET, β-Catenin (Bars 200 µm), and LKB1 (Bar 500 µm). Insets show a detail of the staining (Bars 50 µm). A panel of mouse SCCs showing differentiated (Diff.) and undifferentiated (Undiff.) regions. Immunohistochemistry shows staining for LKB1, β-Catenin (Bars 500 µm),inset (Bars 100 µm), E-Cadherin (Bars 100 µm) and α6-Integrin (Bars 600 µm). Inset at show the loss of α6-Integrin expression in one of the keratinocyte nests (Bar 200 µm) (E) Ki67 and cleaved caspase-3 staining of three different tumors (Bars 200 µm). (F) Western-Blot showing the amount of LKB1 and β-Actin in primary mouse SSC cell lines derived from tumors raised on Hgf Tg; Lkb1 +/− mice. HaCat cells total lysates are used as a control. (G) Western-Blot showing the amount of LKB1and CDKN1A in skin extracts from indicated mice. GAPDH is showed as loading control. (TIF) [file pgen.1004721.s001.tif]

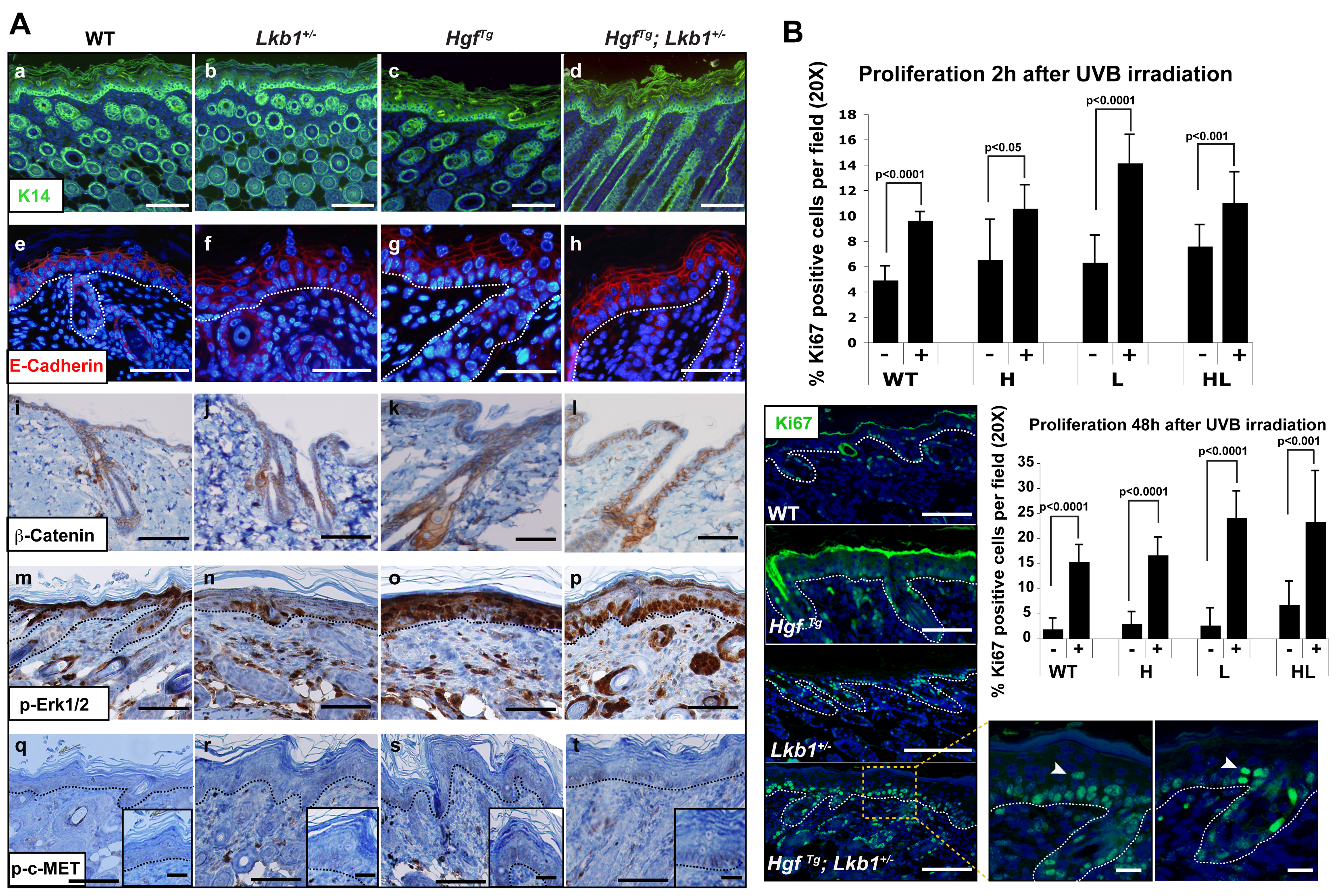

Supplement: Figure S2 — Relate to Figure 1. (A) Keratynocytes differentiation is not compromised neither in the absence of LKB1 or overexpression of HGF. Mouse skin form different genotypes were stained for K14 (a–d),E-Cadherin (e–h), β-Catenin (i–l), p-Erk1/2 (n–p) and p-c-Met (q–t). Representative images are shown. Bars are 400 µm from a–d and j–m; 200 µm e–h, n–q and r–u. (B) Lkb1 +/− and Hgf Tg; Lkb1 +/− mice showed an increased number of keratynocytes recruited into the cell cycle upon UVB irradiation. Bars are 400 µm and 100 µm for magnifications Graphs show quantification of Ki67 positive cells per field 2 hours and 48 hours after UVB irradiation (30 J/m2). At least 30 field/point were evaluated. Error bars represent mean ± SD. P-values were calculated doing a student's t-test. (TIF) [file pgen.1004721.s002.tif]

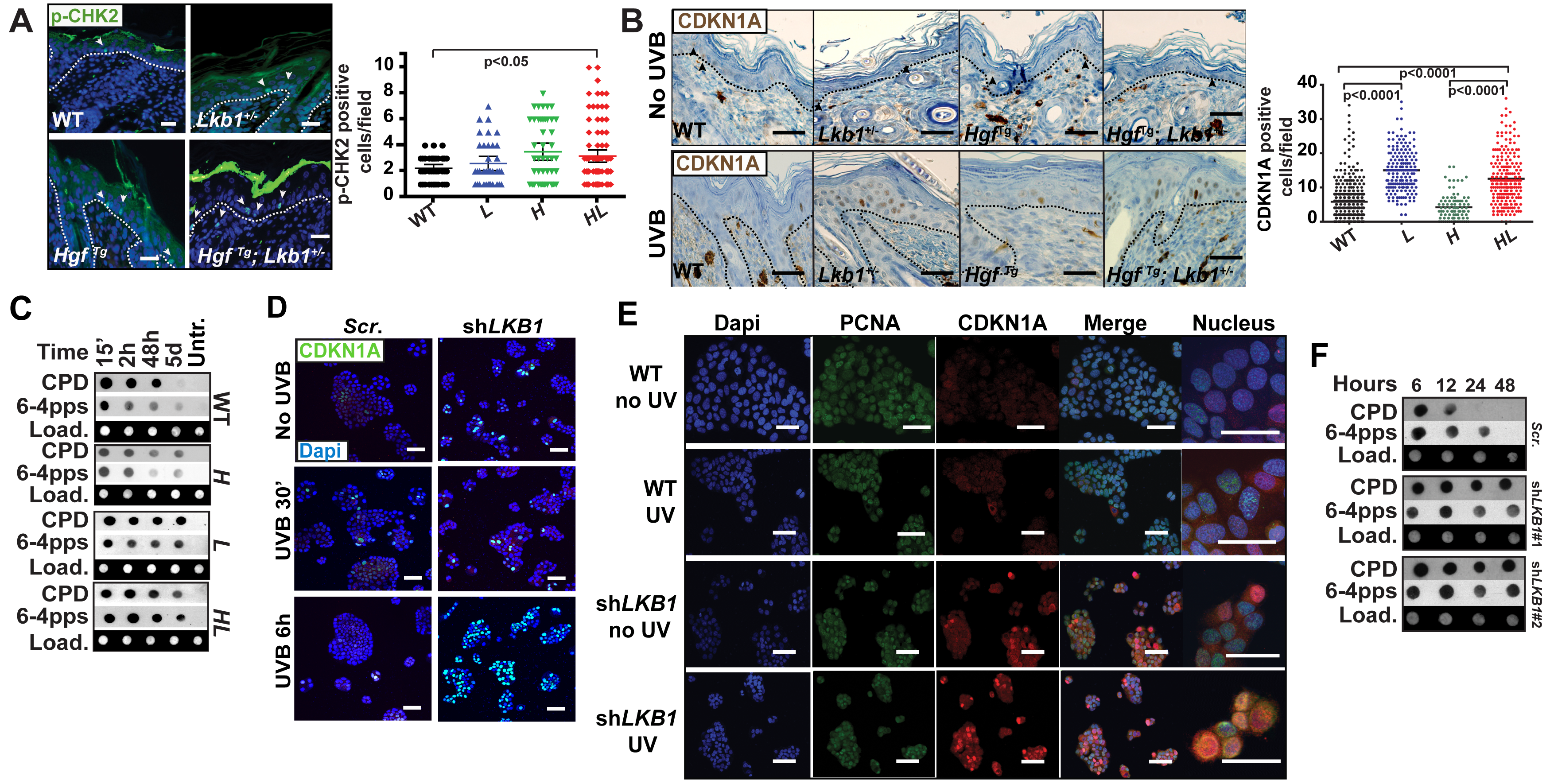

Supplement: Figure S3 — Related Figure 2. Lkb1 haploinsufficiency induces CDKN1A accumulation after UVB-mediated DNA damage. (A) Representative images of mouse skin stained with anti p-Chk2 antibody 48 h after UVB irradiation. Bars 100 µm. Graph shows quantification of p-CHK2 positive basal keratinocytes 48 h post-irradiation. At least fifty fields (20×) from each different mouse genotype (n = 10) were quantified (WT, Lkb1 +/− (L), Hgf Tg (H) and Hgf Tg; Lkb1 +/− (HL)). P-values were calculated using a student's t-test. Error bars represent mean ± SD. (B) Immunohistochemistry of CDKN1A staining showing representative images of mouse skin non-irradiated and 48 h after UVB irradiation. Bars 100 µm. Quantification of CDKN1A positive cells in mouse skin 48 h post-irradiation. At least two hundred and fifty fields (20×) per mouse genotype (n = 10) were quantified. Bars represent mean values. P-values were calculated using a student's t-test. (C) Representative dot blot showing a Global genomic UVB-induced DNA repair analysis performed in skin DNA from WT, Lkb1 +/− and Hgf Tg; Lkb1 +/− mice. Briefly, spotted DNA on the membrane was probed against anti CPD and anti 6-4pps antibodies, as described in experimental procedures. Quantifications of the analysis of at least five mice per genotype and time point are shown in Figure 2 B. (D) Immunofluorescence of CDKN1A in parental infected with Scr. scramble shRNA and LKB1 knocked down HaCat cells (shLKB1#1). One representative experiment out of three is shown. Cells were treated with 30 J/m2 of UVB radiation. Dapi shows nuclear staining. Bars 50 µm. (E) PCNA co-localizes with CDKN1A in the nucleus in response to UVB radiation in LKB1 knockdown cells. HaCat cells and stable LKB1 knockdown cells were treated with UVB (30 J/m2). Then, 6 hours after treatment cells were stained for PCNA and CDKN1A. Dapi was used for nuclear staining. Representative images are shown. Bars = 50 µm. (F) Representative dot blot showing a global genomic UVB-induced DNA repair an [file pgen.1004721.s003.tif]

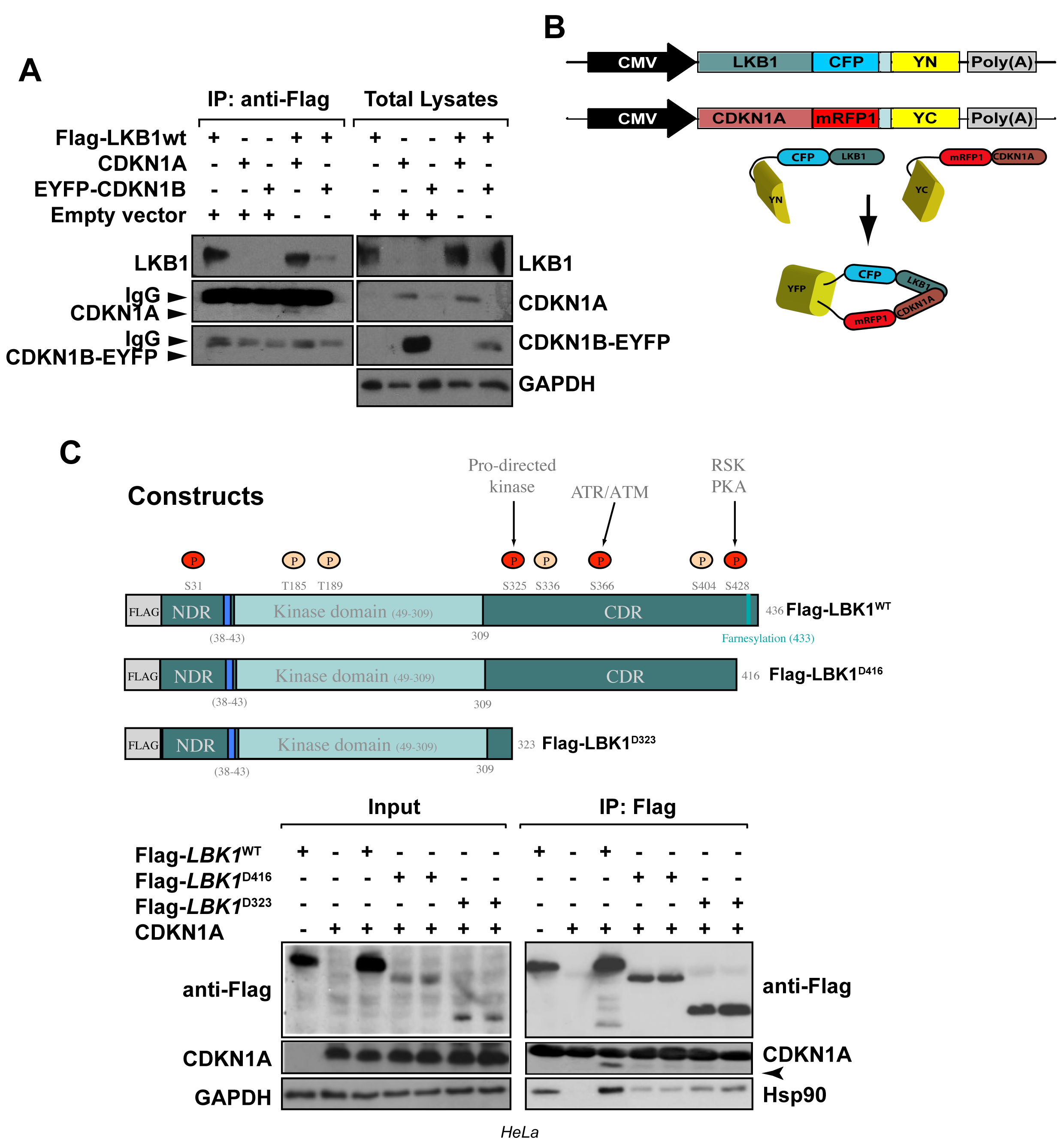

Supplement: Figure S4 — Related to Figure 3. (A) LKB1 binds to CDKN1A but not to CDKN1B (p27). HeLa cells were transfected with equimolar amounts of Flag-Lkb1WT, Flag STRADα and Mo25 together with either CDKN1A or CDKN1B-EYFP (p27). Western-blot shows the presence of CDKN1A and CDKN1B proteins bound to LKB1. Total lysates are shown for transfection control. (B) Constructs for BiFC assay, Lkb1-CFP-YFPN and CDKN1A-mRFP1-YFPC constructs were transfected in HeLa cells, in the absence or presence of Flag-STRADα and MO25α (figure 3D). (C) Schematic representation of LKB1 deletion mutants. HeLa cells were transfected with either the different LKB1 constructs together with CDKN1A. LKB1 was immunoprecipitated using anti Flag antibody. After SDS-PAGE the amount of CDKN1A bound to LKB1 was assessed by western-blot. HSP90 is showed as a positive control binding protein for LKB1. (TIF) [file pgen.1004721.s004.tif]

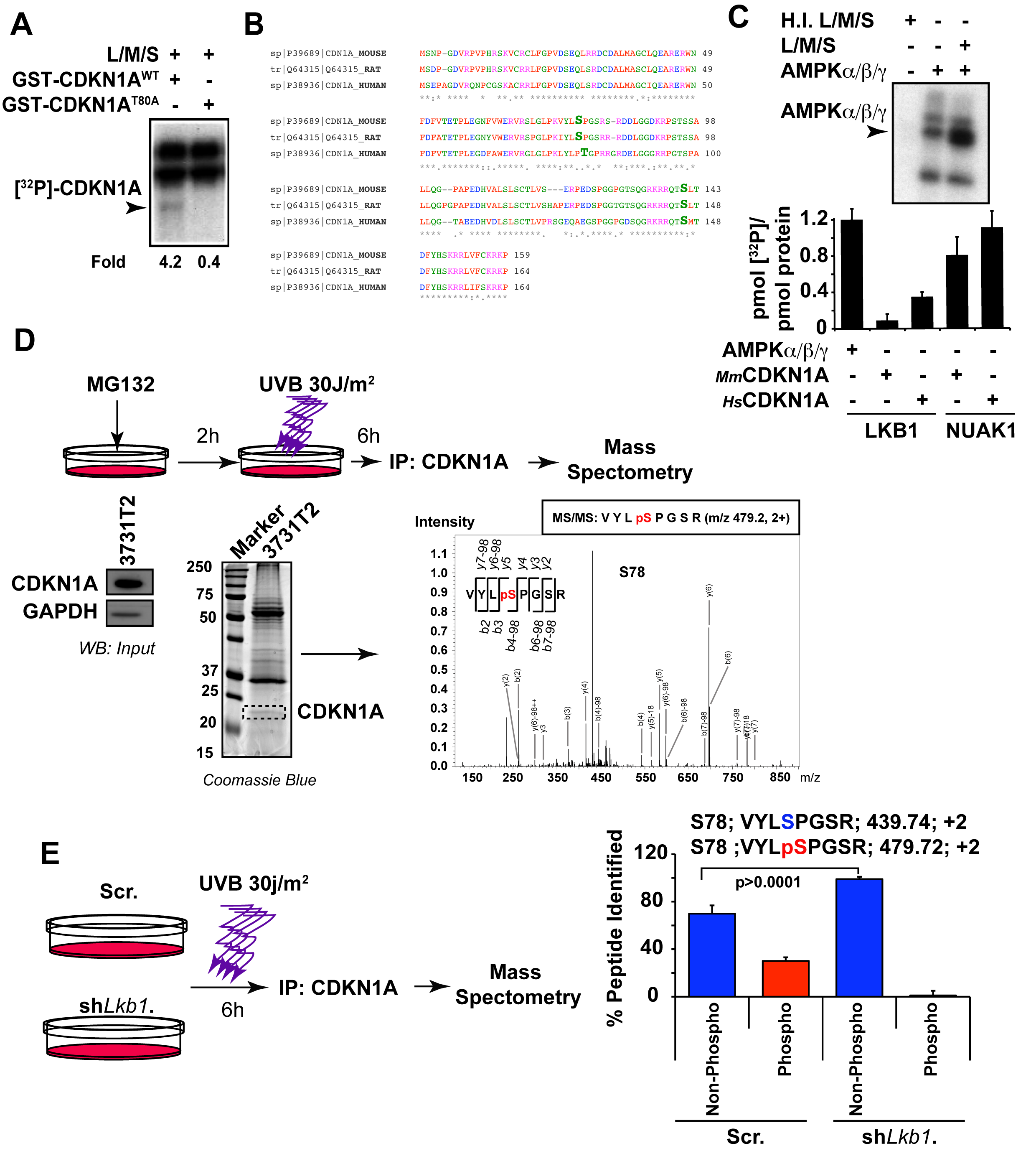

Supplement: Figure S5 — Related to figure 4. LKB1 mediates mouse CDKN1A phosphorylation in response to UVB. (A) In vitro kinase radioactive assay of LKB1:STRAD:MO25 (250 ng) using human recombinant GST-CDKN1A and GST-CDKN1AT80A mutant as a substrate (300 ng). Number below shows differences in fold changes in GST-CDKN1A phosphorylation. (B) Mouse and Human sequence alignment. Human T80 and Mouse S78 appear highlighted. (C) In vitro kinase assay of LKB1:STRAD:MO25 (250 ng) using AMPKα/β/γ as a substrate (300 ng). Pmol of phosphate incorporated per pmol of substrate by either LKB1 or NUAK1 is shown in the graph. All the experiments were performed in triplicates. Error bars represent mean± SD. (D) 37-31T2 mouse melanoma cells were treated with MG132 (200 nM) for 2 h and then irradiated with UVB (30 J/m2). Cells were lysed six hours post irradiation. Western-blot showing the accumulation of CDKN1A. Then CDKN1A was immunoprecipitated. Gel shows CDKN1A amounts after coomassie blue staining. Bands corresponding to CDKN1A size were analyzed by mass spectrometry. Fragmentation spectrum of mouse protein peptide containing S78 is shown. (E) 37-31T2 cells infected with scrambled (Scr.) or shLkb1 were treated as in (D) followed by mass spectrometry of immunoprecipitated CDKN1A percentage of non-phosphorylated and phosphorylated peptides at residue S78 is shown in the graph. Error bars represent mean ± SD. P-value were calculated using a student's t-test. (TIF) [file pgen.1004721.s005.tif]

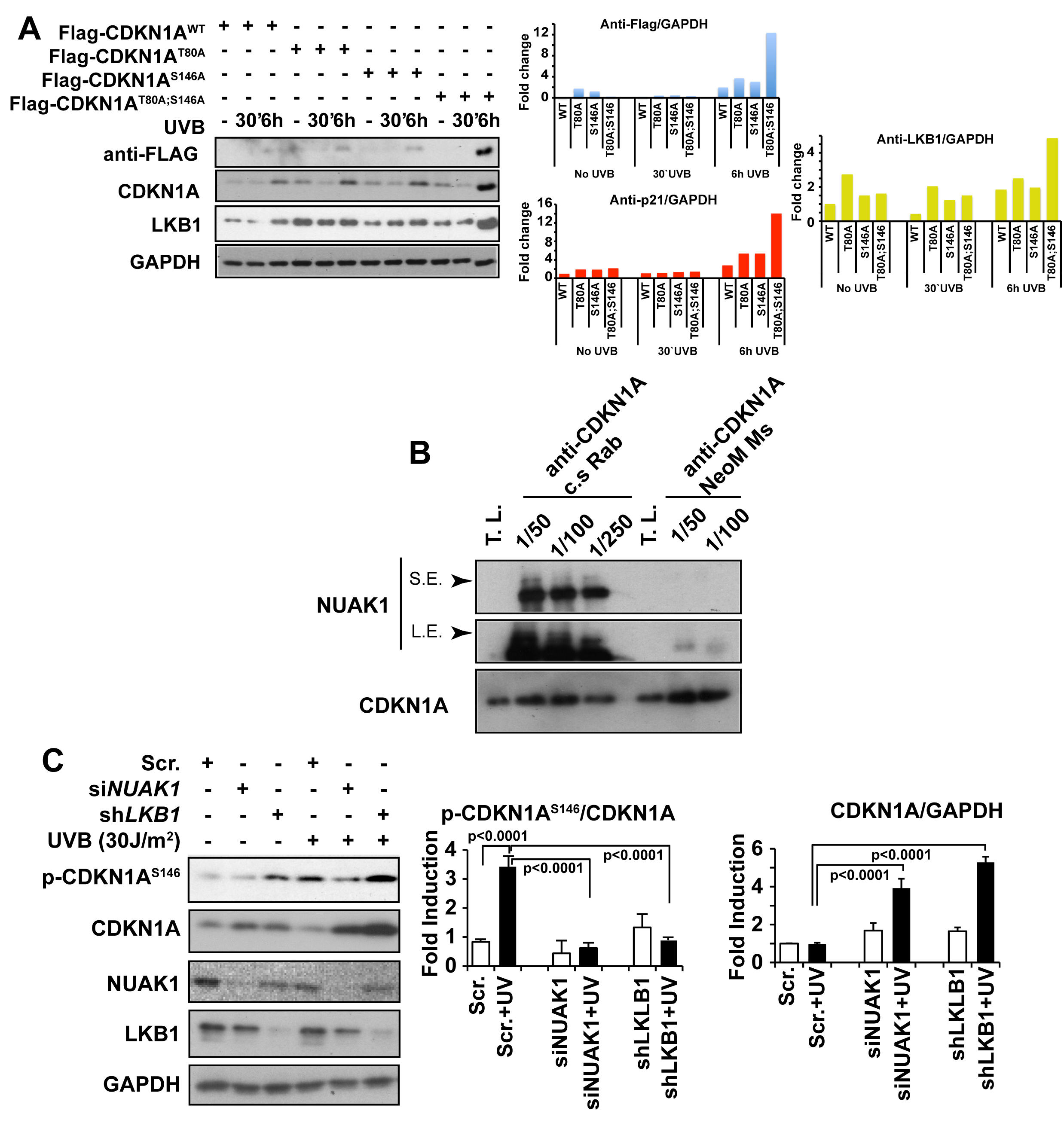

Supplement: Figure S6 — Related to figure 4. CDKN1A phosphorylation site mutants T80A, S146A and T80A; S146A are accumulated in responses to UVB irradiation. (A) HaCat cells were transiently transfected with wild type and mutant isoforms of CDKN1A. Then cells were UVB irradiated (30 J/m2) and lysed after 30 minutes and 6 h. Western blot shows the amounts of CDKN1A, LKB1 and GAPDH. Graph shows normalized quantification against GAPDH. One representative experiment out of three is showed. NUAK1 and CDKN1A form part of the same immunocomplexes. 37-31T2 mouse melanoma cells were treated with MG132 (200 nM) for 2 h and then irradiated with UVB (30 J/m2). Cells were lysed six hours post irradiation. (B) Two different antibodies against p21WAF1/CIP1were used to immunoprecipitate CDKN1A at the indicated dilutions. Western-blots show the amount of CDKN1A immunoprecipitated and the amount of NUAK1 present in the immunocomplexes. (C) HaCat cells transiently transfected with either NUAK1 siRNA or scrambled siRNA and HaCat cells stable infected with shLKB1 were irradiated with 30 J/m2 of UVB. Total protein lysates were analyzed by SDS-PAGE 6 h post-irradiation. Amounts of p-CDKN1ASer146, p21WAF1/CIP, NUAK1, LKB1 and GAPDH are shown. Graphs show the amounts of p-CDKN1ASer146 relative o the amount of CDKN1A and the amounts of CDKN1A relative to the amount of GAPDH. P- values were calculated performing a student's t-test. (TIF) [file pgen.1004721.s006.tif]

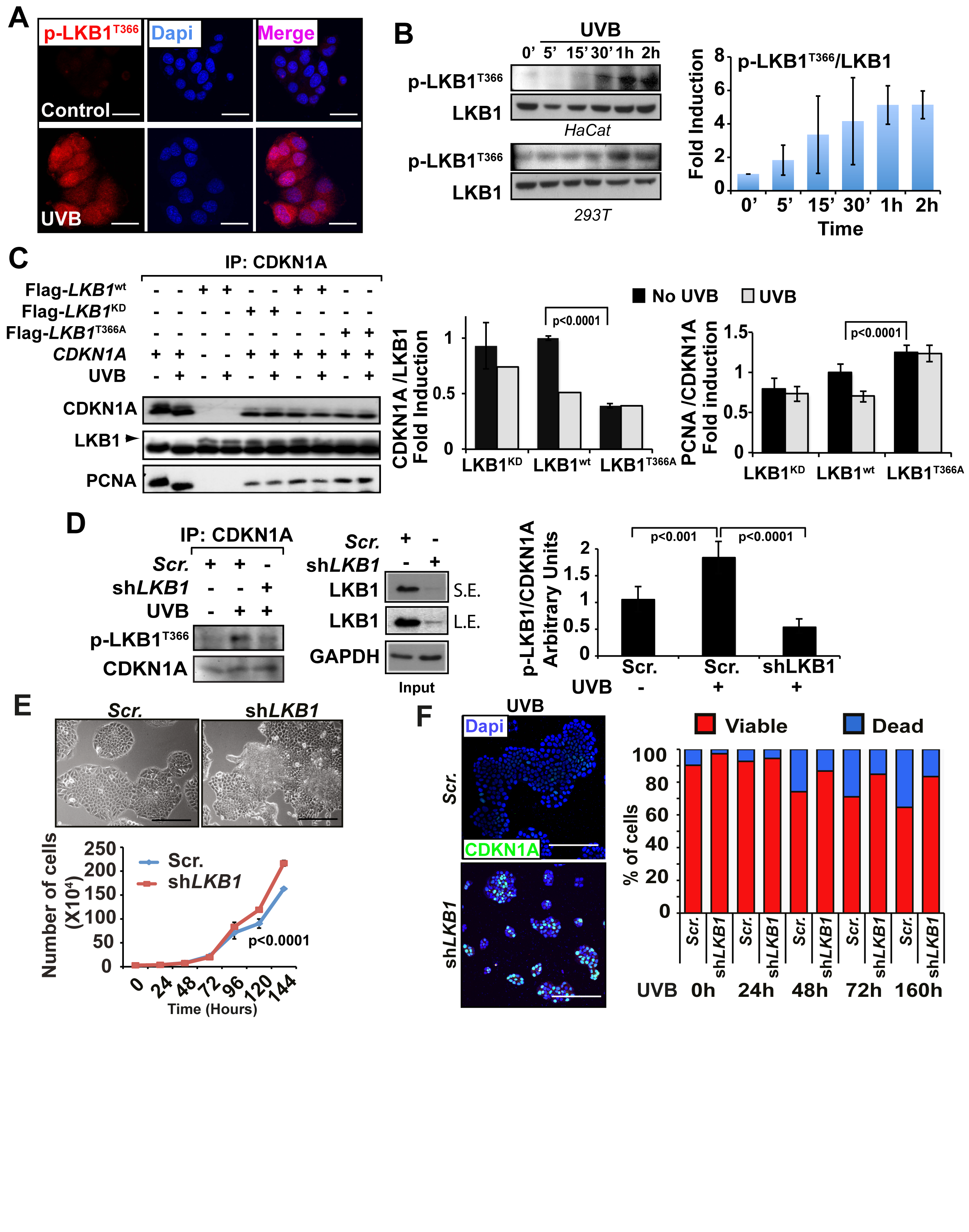

Supplement: Figure S7 — Related to figure 5. UVB-induced phosphorylation of LKB1T366 is involved in the binding to CDKN1A. (A) Representative pictures (n = 3 experiments) of immunofluorescence of p-LKB1T366 in HaCat cells 4 h after UVB irradiation. Dapi shows nuclear staining. (B) HaCat and 293T cells were irradiated with 30 J/m2 of UVB (n = 3 experiments). Samples were analyzed by western-blot at the times indicated. The amount of p-LKB1T366 relative to the amount of LKB1 is shown. Quantification of p-LKB1T366 relative to the amount of LKB1 in the time course is shown. (C) Total lysates from (Figure 5 A) were used to immunoprecipitate CDKN1A. Western-blot shows the amount of Lkb1 and PCNA in the immunocomplexes. Graphs on the right show the quantification of LKB1 and PCNA bound to CDKN1A (n = 3 experiments). (D) CDKN1A was immunoprecipitated from HaCat cells transfected either with scrambled shRNA or shLKB1#1 6 h after UVB irradiation (30 J/m2). Western-blot shows the abundance of p-LKB1 bound to CDKN1A. Graph shows the ratio of p-LKB1T366 bound to CDKN1A under the different conditions. One representative experiment out of three is shown. Error bars represent mean ± SD. P-value was calculated performing a student's t-test. Related to Figure 6. Depletion of LKB1 promotes pro-tumorigenic features and resistance to UVB radiation. (E) HaCat cells stably infected with shLKB1 showed an increased proliferation and lost cell-cell contact inhibition. Representative images using one of the three different shLKB1 are shown. Bars are 200 µm. (F) HaCat cells infected either with scrambled or shLKB1 were irradiated with UVB (30 J/m2). Representative images of cells stained against CDKN1A 10 h post-irradiation are shown. On the right number of viable and dead cells were quantified at different time points by nuclear staining exclusion (Guava-ViaCount). (TIF) [file pgen.1004721.s007.tif]

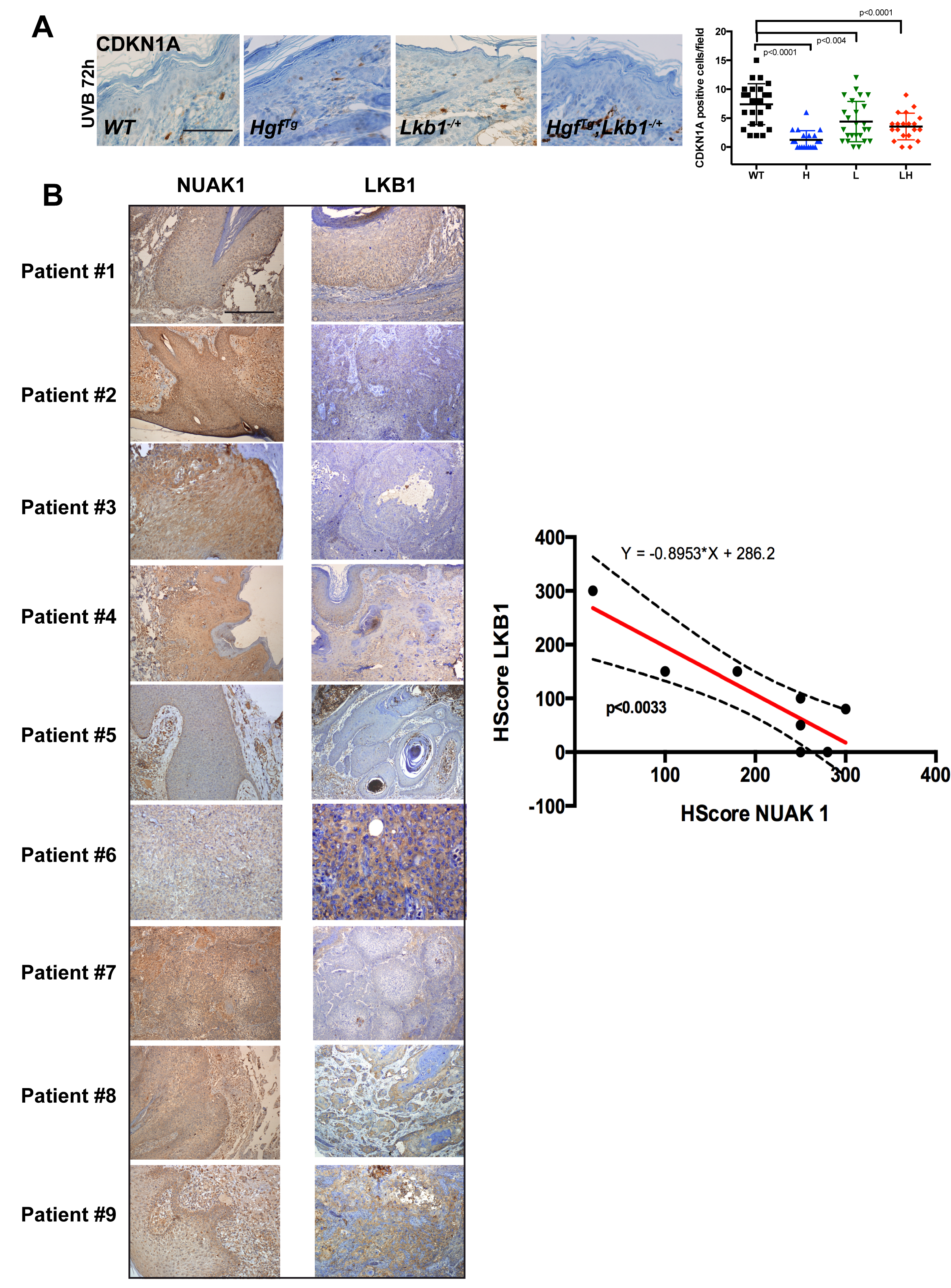

Supplement: Figure S8 — (A) Expression of CDKN1A in mouse skin after 72–80 h after UVB irradiation. Quantification of positive cells of at least 20 fields (20×) per genotype (three different mice) is showed on the right. Bar represent 100 µm. (B) Staining of same tumor samples for LKB1 and NUAK1. Correlation of the LKB1 and NUAK1 HScore for each sample is plotted on the right. For this plot sample from patient #5 staining negative for both proteins was excluded. 95% confidence interval is showed in the graph. Bar represent 200 µm. (TIF) [file pgen.1004721.s008.tif]

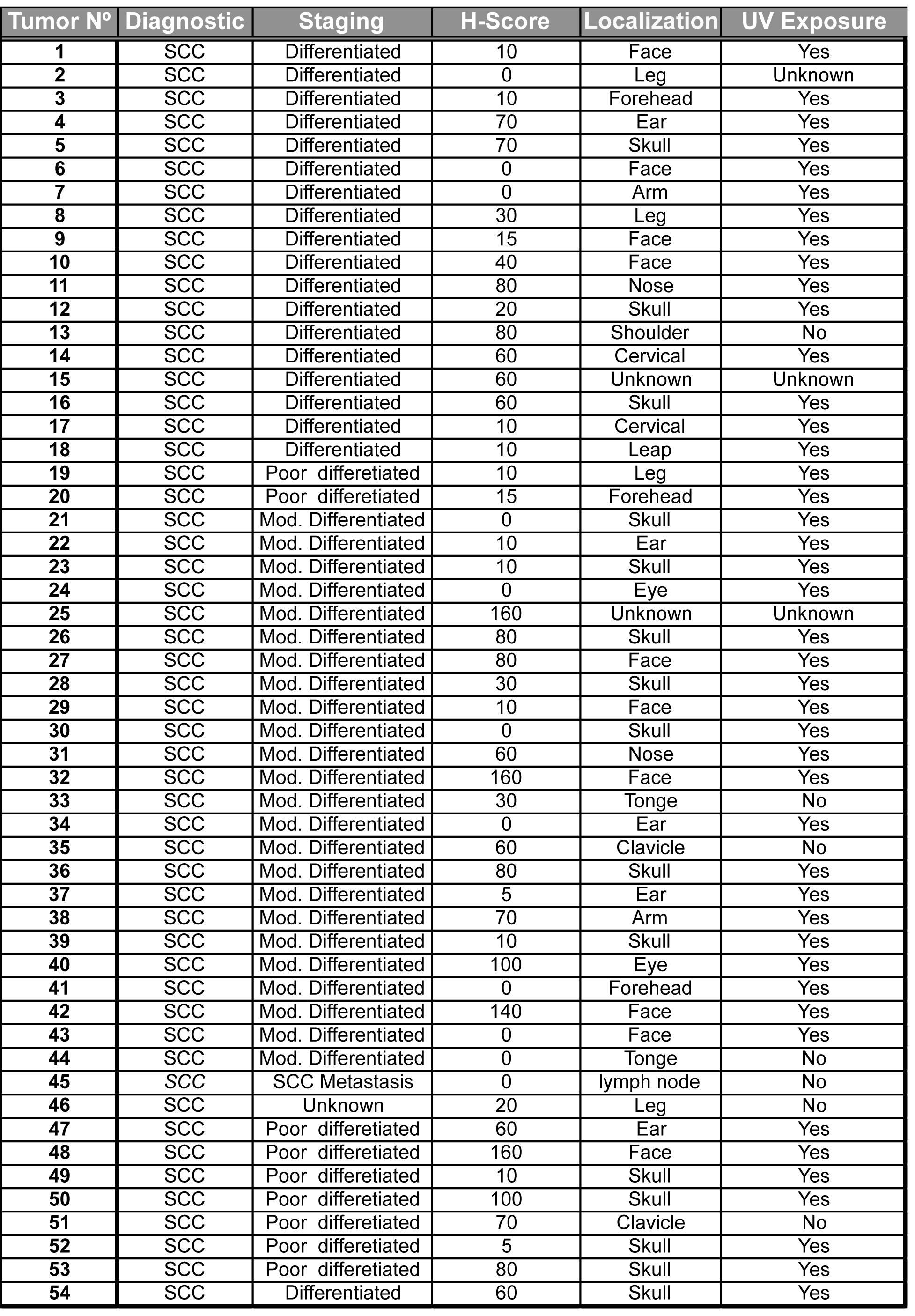

Supplement: Table S1 — Related to Figure 6. Human tumor samples used in the study. Diagnostic, grade of differentiation (staging), Hscore, anatomical localization and UV exposure component are shown. (TIF) [file pgen.1004721.s009.tif]
